# Exploring the role of nanomedicines for the therapeutic approach of central nervous system dysfunction: At a glance

**DOI:** 10.3389/fcell.2022.989471

**Published:** 2022-09-02

**Authors:** Md. Mominur Rhaman, Md. Rezaul Islam, Shopnil Akash, Mobasharah Mim, Md. Noor alam, Eugenie Nepovimova, Martin Valis, Kamil Kuca, Rohit Sharma

**Affiliations:** ^1^ Department of Pharmacy, Faculty of Allied Health Sciences, Daffodil International University, Dhaka, Bangladesh; ^2^ Department of Chemistry, Faculty of Science, University of Hradec Králové, Hradec Králové, Czech Republic; ^3^ Department of Neurology, Charles University in Prague, Faculty of Medicine in Hradec Králové and University Hospital, Hradec Králové, Czech Republic; ^4^ Andalusian Research Institute in Data Science and Computational Intelligence (DaSCI), University of Granada, Granada, Spain; ^5^ Department of Rasa Shastra and Bhaishajya Kalpana, Faculty of Ayurveda, Institute of Medical Sciences, Banaras Hindu University, Varanasi, India

**Keywords:** neurodegenerative diseases, blood-brain barrier, drug delivery, nanotechnology, nanomedicine and nanocarrier

## Abstract

In recent decades, research scientists, molecular biologists, and pharmacologists have placed a strong emphasis on cutting-edge nanostructured materials technologies to increase medicine delivery to the central nervous system (CNS). The application of nanoscience for the treatment of neurodegenerative diseases (NDs) such as Alzheimer’s disease (AD), Parkinson’s disease (PD), multiple sclerosis (MS), Huntington’s disease (HD), brain cancer, and hemorrhage has the potential to transform care. Multiple studies have indicated that nanomaterials can be used to successfully treat CNS disorders in the case of neurodegeneration. Nanomedicine development for the cure of degenerative and inflammatory diseases of the nervous system is critical. Nanoparticles may act as a drug transporter that can precisely target sick brain sub-regions, boosting therapy success. It is important to develop strategies that can penetrate the blood–brain barrier (BBB) and improve the effectiveness of medications. One of the probable tactics is the use of different nanoscale materials. These nano-based pharmaceuticals offer low toxicity, tailored delivery, high stability, and drug loading capacity. They may also increase therapeutic effectiveness. A few examples of the many different kinds and forms of nanomaterials that have been widely employed to treat neurological diseases include quantum dots, dendrimers, metallic nanoparticles, polymeric nanoparticles, carbon nanotubes, liposomes, and micelles. These unique qualities, including sensitivity, selectivity, and ability to traverse the BBB when employed in nano-sized particles, make these nanoparticles useful for imaging studies and treatment of NDs. Multifunctional nanoparticles carrying pharmacological medications serve two purposes: they improve medication distribution while also enabling cell dynamics imaging and pharmacokinetic study. However, because of the potential for wide-ranging clinical implications, safety concerns persist, limiting any potential for translation. The evidence for using nanotechnology to create drug delivery systems that could pass across the BBB and deliver therapeutic chemicals to CNS was examined in this study.

## Introduction

The World Health Organization categorizes central nervous system (CNS) malignancies based on their effective potential, likelihood of spread, and clinical prognosis (Louis et al., 2016; Singh et al., 2021a). Because the brain accounts for nearly all malignant CNS tumors, the focus will now be on the lookout for malignant brain tumors. Malignant brain tumors are a type of primary brain tumors; for instance, almost 2% of all cancers and are emblematically generated from glial cells (hence the name gliomas) (Miranda et al., 2017). Brain metastases affect 10%–30% of all cancer patients, with 70%–80% developing numerous injuries. The high rate of intracranial metastases is due to the fact that while new chemotherapeutic drugs have transformed prognosis for many forms of cancer, they have been able to deter neoplasms from spreading into the brain due to their limited blood–brain barrier (BBB) penetration. The invention of therapeutic approaches that can prevent the BBB and also increase their efficacy is required. To address this issue, researchers are actively developing therapeutic drug delivery techniques (Parveen et al., 2012a; Zhang et al., 2015; Upadhyay, 2014). Different approaches to solve this issue have been developed. The nano-based method is at the heart of recent advancements in therapeutic drug delivery (Sharma et al., 2019; Song et al., 2021). Different kinds of manufactured nanomaterial and nanoparticles with a size of 1–100 nm in at least one dimension are used in nanotechnology (Farokhzad and Langer, 2009; Sharma and Bhargava, 2013; Khan et al., 2016). As numerous nanoparticles have been used in brain studies and research, including quantum dots (QDs), polymeric nanoparticles, micelles, and metallic nanoparticles, nanotechnology and nanomaterials open new pathways in biomedical science (Chenthamara et al., 2019; Alshamrani, 2022; Waris et al., 2022). Because of their small size and capacity to interact with biological systems at the molecular level, these nanoscale materials also have special properties such as a high surface-to-volume ratio that can be mono or diverse with surface modification and also high stability (Umut, 2013; Natarajan et al., 2019; Singh et al., 2020). This is a significant impediment to brain delivery (Au et al., 2017). In malignant brain tumors, the mainstay of care is maximal surgical resection (if possible), followed by radiation, chemotherapy, and symptomatic treatment (Alifieris and Trafalis, 2015). Nonetheless, since reappearance within the next few times is typical, cancerous brains tumors are prone to relapse (brain metastases have quite a median survival of 8 months, even aggressive primary brain cancers have median survival of 14.2 months) and remain an unmet clinical challenge (Stupp et al., 2005; Au et al., 2017). Indeed, given the extraordinarily varied character of malignant brain tumors, it is no longer reasonable to expect a single treatment to be effective in all individuals. At most, each medication would be helpful only for specific target populations and disease stages. In this context, theragnostic, which is defined as the simultaneous delivery of imaging and therapeutic substances (Orive et al., 2010), has a lot of potential for the treatment of malignant brain tumors at various stages. A number of promising medications for the treatment of neurological diseases have been identified (Alguacil et al., 2003; Whiting, 2003; Langmead et al., 2008). Although these medications have shown therapeutic efficacy, the existence of the 1) BBB and the 2) blood–cerebrospinal fluid barrier (BCFB) continues to restrict and limit their effectiveness (Jain, 2007; Wong et al., 2012; Dominguez et al., 2014). A number of scientists are trying to develop an associative strategy using nanotechnology in order to get the better at these significant challenges toward the field of CNS therapy. In the future, nanoparticles and their complex mixture including therapeutic substances may be considered as an effective tool in brain medication delivery for the development of better medicines (Halberstadt et al., 2006; Jain, 2007; Singh et al., 2019a; Singh et al., 2019b). For the treatment of CNS diseases, nanoengineered materials are important and helpful for a number of reasons. The materials can first and foremost penetrate the BBB, which is a frequent barrier to CNS-targeted medicines (Srikanth and Kessler, 2012; Ai et al., 2016). In addition, nanomaterials can be created to interact with specific cellular subsets or chemicals, allowing for more targeted treatment. Also, the nanomaterial’s incorporation of enzyme cleavage sequences permits modulation of activity in response to biological stimuli, such as pH-sensitive modification or cation-triggered self-assembly (Johnstone et al., 2016; Karamanos et al., 2018; Rai et al., 2021). For either endogenous or transplanted cells, nanofibers and nanoscaffolds can offer trophic support in addition to structural support. The fact that nanoengineered materials can include many properties into their structures to simultaneously provide targeting, bioactivity, gene transport, and imaging capabilities in a single material is significant (Srikanth and Kessler, 2012; Liu J. et al., 2015; Bhatia, 2016). We discussed the therapeutic significance of nanomedicine in CNS dysfunction. In this review, we give an overview of the nanotechnologies that have been studied in relation to neurological disease, talk about the evidence for effectiveness and toxicity of nanomaterials in particular CNS disorders, and emphasize the potential for clinical application of nanotechnology.

## Blood–brain barrier

**FIGURE 1:**
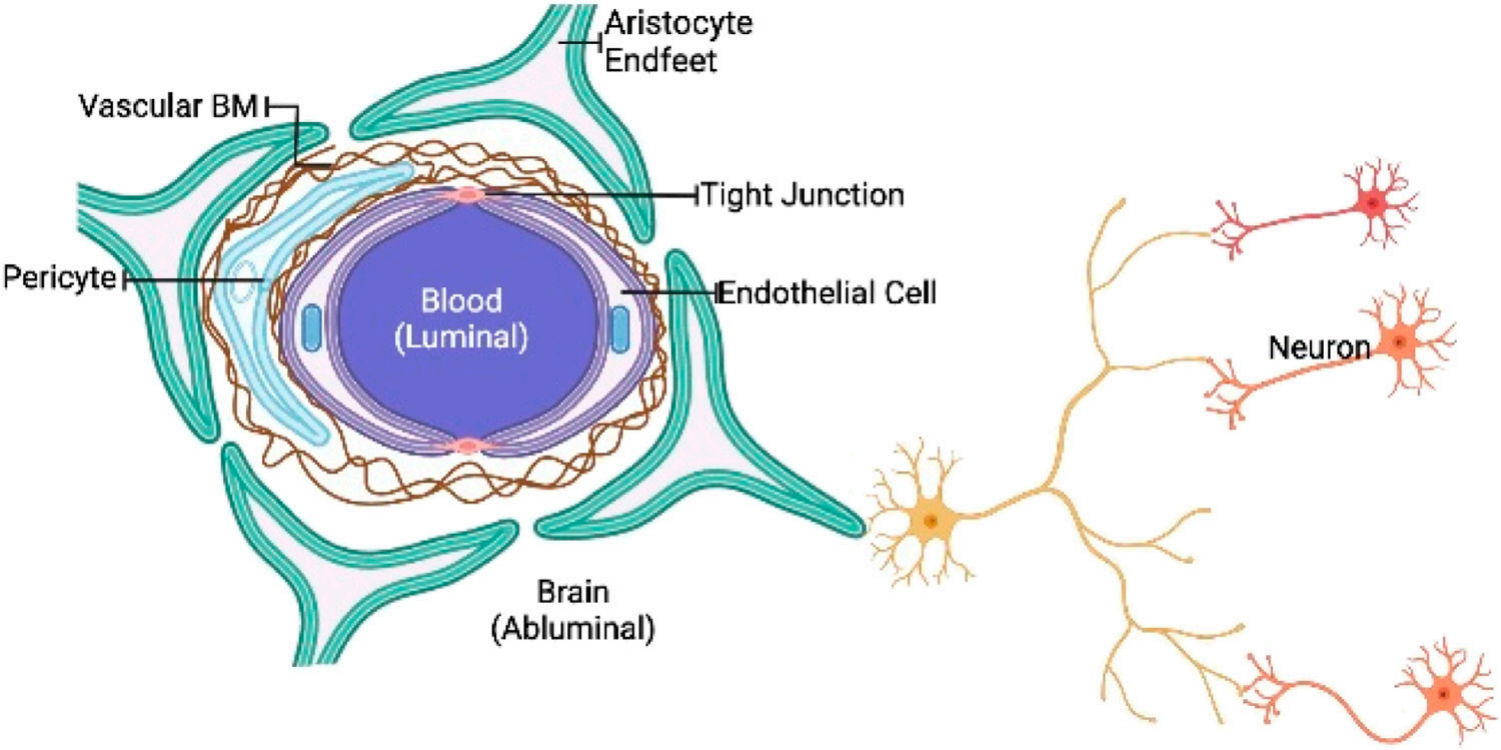
Schematic representation of BBB. The BBB is a highly biased semipermeable barrier of endothelial cells that blocks the non-selective movement of substances in the bloodstream into the extracellular fluid of the central nervous system, where neurons reside.

The brain is thought to be the body’s most safeguarded organ. The skull, peripheral nerves, cerebrospinal fluid, and BBB are all protective shields for the central nervous system. Many of these elements have the capacity to prevent the brain against internal and external traumas as well as disease prevention (Engelhardt and Sorokin, 2009; Nair et al., 2018). These protective barriers, however, reduces of entry therapeutic agents to the central nervous system in diseased states. The BBB is a physiological border made up of firmly bound cerebral vascular wall, hepatocytes, astrocytes, and basal membranes, with occasional endocytosis and transcytosis (Vorbrodt and Dobrogowska, 2003; Yazdani et al., 2019). Most medicines are rapidly effluxed due to the high expression of P glycoproteins on brain vascular endothelium (Andersson et al., 2002; Miller, 2010). The BBB blocks practically all polymers and a major proportion of tiny molecules (especially chemotherapy drugs) from reaching the CNS, especially the brain, because of relatively narrow openings in such connecting cells (Domínguez et al., 2013; Karim et al., 2016; Furtado et al., 2018). As a result, the BBB is blamed for the inadequacy of most treatment options. Epithelial lymphocytes and membranes structure such as vascular endothelium and mucosal membranes have the capacity to transfer materials into the CNS because of their lipid-like composition (Figure 1) (Alam et al., 2010; Yu et al., 2016).

## Drug targeting

Researchers are focusing on specific areas inside the target cell to help drugs accumulate at the target spot and reduce side effects. Most nanoparticles created to date have failed to deliver drug material to a particular sub-cellular target organelle. The sub-cellular localization of nano-particulate therapeutic compounds administrate applications is still under progress (D’Souza and Weissig, 2010). Organelle-specific targeting sites, such as TAT CPP (cellular permeating peptides), energy metabolism targeted identification, nanomaterial surfaces, target-specific cell organelles, and nuclear clustering signals are all used to their full capability for drug delivery (Flierl et al., 2003; De la Fuente and Berry, 2005; Kang et al., 2010). As a result, sub-cellular targeting could dramatically minimize the amount of therapeutic molecule required.

### Endosomal targeting

Nanotherapeutics might be given to lysosomes or endosomes by receptor-specific endocytosis or by engaging to the target site on the endosomal cellular region; moderate lipid membranes, ferritin, and ventricular endothelium proliferation considerations are examples of these substances. The endosomes are also responsible for a fundamental function in nanoparticle preservation by delaying depreciation so that they can continue to function in the cytoplasm (Sakhrani and Padh, 2013).

### Endoplasmic reticulum–specified targeting

Golgi bodies are secretory cell organelles. They engage with endoplasmic reticulum (ER), a folded membrane network that extends from the nuclear membrane throughout the cytoplasm. The ER is associated within Ca^2+^ stockpiling, Ca^2+^ signaling, apoptosis regulation, and facilitating the folding of secretory proteins. Golgi bodies use a variety of enzymes to modify newly generated proteins after they have been translated. mTOR (mammals or mechanical mediator of starvation) is a proteolytic enzyme that plays a role in cell signaling that is mostly found in the ER and Golgi bodies. Cellular differentiation modulation, polypeptide hydrolysis, PCR amplification, protein interactions, phagocytosis, and cell function are all maintained by it, among other things (Lu et al., 2018; Sanati et al., 2019).

### Mitochondrial targeting

A mitochondrion is known as a cell’s powerhouse because it supplies ATP (Adenosine Triphosphate) for energy generation through oxidative phosphorylation process. It also removes ROS in a variety of ways. ROS are created as by-products of ATP synthesis, which can lead to mitochondrial malfunction. In the case of AD, this could interact with the peptides, causing amyloid buildup in the brain. The mitochondria have certain antioxidants, such as MitoQ and SS31, which are used to treat oxidative stress and synaptic dysfunction in AD (McManus et al., 2011).

### Nuclear targeting

The nucleus, cell’s control center, holds genetic material, making it vital because it must regulate expression of gene for protein creation, which is specifically dispersed within the nucleus and cytoplasm. Nuclear pore complexes (NPC) are pores in the bilayer membrane that divide the nucleus from the cytoplasm that allow for this translocation. NPC-targeting nanoparticles can be delivered into the nucleus (Xia et al., 2016; Popli et al., 2018).

### Organic nanoparticle

Due to their biocompatibility, biodegradability, and overall non-toxicity, a broad range of distinct organic nanoparticles (Table 1) have been explored as vaccination platforms (Poon and Patel, 2020). In comparison to other nanoparticle platforms, organic nanoparticles have many benefits, such as the ability to self-assemble antigens and adjuvants under physiologically mild conditions, as well as chemical uniqueness to accommodate various methodologies, mixtures, dimensions, shapes, and surface modification (Poon et al., 2018; Poon and Patel, 2020; Zhang et al., 2021).

**TABLE 1:** Organic and inorganic vaccine nanoparticles against infectious diseases.

Nanoparticles	Antigens	Shape	Size (nm)	Diseases	References
Organic					
Polymeric	Hepatitis B surface antigen	Spherical	474–940	HBV	Thomas et al. (2011)
	VMP001	Spherical	290	Malaria	Aikins et al. (2017)
Liposome	Hemagglutinin of IAV and IBVa	Spherical	50–400	IAV and IBV	(Glück et al., 1994; Patois et al., 2012)
	Membrane-proximal external region (MPER) peptide	Spherical	150	HIV	Hanson et al. (2015)
Virus-like particle	HPV16 L1 capsomeresa	Pentameric	10	HPV	Hassett et al. (2015)
Inorganic					
Gold	West Nile virus envelope protein	Spherical, rod, and cubic	20–40 (spherical) 40 × 10 (rod) 40 × 40 **×** 40 (cubic)	West Nile virus	Niikura et al. (2013)
Iron Oxide	M. tb fusion protein	Spherical	<20	M. tb	Pusic et al. (2013)
	Mannose and HBsAg	Spherical	60	HBV	Rezaei et al. (2019)
Mesoporous Silica	Soluble worm antigenic preparation antigen	Spherical	39	*Schistosoma mansoni*	de Pádua Oliveira et al. (2016)
	Porcine circovirus type 2 opening reading frames (PCV2-ORF2) proteins	Spherical	200	Post-weaning multisystemic wasting syndrome	Guo et al. (2012)

### Inorganic nanomaterials

Inorganic nanoparticles (Table 1), also known as INPs, have been the subject of research over the period of the several years for a broad range of different commercial applications. INPs have been put to use in the area of biomedicine for the goals of diagnosis as well as treatment (Lohse and Murphy, 2012; Giner-Casares et al., 2016). For instance, gold nanoparticles (AuNPs) have been the subject of a deep investigation along with their biomedical applications and relieve with which their particle sizes and shape can be controlled. Their structure can take on a variety of forms, including spheres, nanorods, and cubes, among others (Gurunathan et al., 2014; Singh et al., 2016). In addition, the surface chemistry of AuNPs is able to be readily changed by combining with a wide variety of polymers, antibodies, small-molecule therapies, and molecular diagnostics (Lee et al., 2010; Zhang et al., 2020). This part will highlight new advancements in Inorganic and organic nanoparticle vaccine delivery platforms, such as, gold, mesoporous silica, iron oxide, polymeric nanoparticles, liposomes, and virus-like particles (VLPs).

### Current FDA approved synthetic drugs for CNS dysfunction

The central nervous system, which comprises nerves located in both the brain and the spinal cord, is the part of our body that processes and regulates the vast majority of our biological activities (Elenkov et al., 2000; Rossini et al., 2015). Medicines that have an effect on the central nervous system or may trigger CNS are known as central nervous system medications (Misra et al., 2003). A variety of medications, such as sedatives, antidepressants, and anesthetics, are all examples of medications that may affect the central nervous system (Charney et al., 2006; Henschel et al., 2008; Attri et al., 2012). Details are given in the table (Table 2)**.**

**TABLE 2:** Synthetic drugs acting on the CNS and their possible limitation.

Therapeutic class	Pharmacological Use	Limitation/Possible side effect	Examples	References
Antidepressants	CNS stimulants; anticholinergic	Tricyclics may promote dry mouth, impaired vision, tachycardia, and cardiac arrythmias	Monoamine oxidase inhibitors and tricyclic antidepressants	(Walsh, 1979; Livingston and Livingston, 1996; Youdim et al., 2006)
Antipsychotics	Relieve anxiety and thought disturbances	These medications have the potential to induce drowsiness, hypothermia, hypotension, and lowering in seizure threshold.	Butyrophenones and phenothiazines	(Janssen, 1965; Martin et al., 1992)
Antiemetics	Relieve nausea and vomiting	Antihistamines are the only treatments that are recommended for divers to use in order to prevent motion sickness; nevertheless, the sleepiness generated by these medications might induce a reduction in cognitive ability.	Anticholinergics and antihistamines	Williams et al. (1988)
Anxiolytics	Relieve anxiety; depress CNS	Anxiolytic medications often produce sleepiness, lethargy, disorientation, and hypotension, all of which have the potential to be catastrophic in the water.	Benzodiazepines	(Groner-Strauss and Strauss, 1976; Greenblatt et al., 1983; Stewart, 2005)
CNS Stimulants	ncrease alertness; inhibit fatigue; suppress appetite; mood elevation	These may bring on symptoms such as exhilaration, increased perspiration, anxiety, and panic attacks.	Amphetamines	(Cheshire et al., 2001; Fleckenstein et al., 2007; Carvalho et al., 2012)
Hypnotics	Depress CNS and induce sleep	It may produce drowsiness, asthenia (weakness), headache, and aeuromuscular and skeletal weakness	Barbiturates	(Vermeeren, 2004; Charney et al., 2006)

## Modeling techniques for CNS disorders

Sickness modeling in the lab is essential for improving comprehension of pathophysiology and evaluation of efficiency of innovative pharmacological strategies. Cancerous cells can come from a variety of places: cancer tissues or tumor cell culture generated in the laboratories, which are mainly derived from transgenic mice or from patients (Liu F. et al., 2015; Humpel, 2015). Brain or systemic infusions of created cancer cells into recipient mice are used to generate multiple myeloma characteristics in desired experimental animals (Verreault et al., 2016). Submucosal or cerebral infusion of cancerous cells into rats is the most extensively used approach in laboratory animals. Transgenic models administered with a dermal injection continue to grow but are not well restricted within the intramuscular region, rendering this model distinct from the TME (Verreault et al., 2016). Intracerebral infusion for tumor transplantation is preferable because cancer cells are directly implanted into the brains rather than being degraded *via* subcutaneous administration (Hoffman, 2015). Other methods of mutant hereditary materials supply, such as chromosomal- and viral carrier (retroviral administration of genomic material)-based template matching delivery of materials (Bardella et al., 2016; Miyai et al., 2017; Oldrini et al., 2018), can also be used to create models. In addition to this, the desired mice should be produced by integrating mice that have mutated genes and the progression of neurological disorders in humans should be triggered. Till now, the majority of AD lab animals have been genetic pigs in which transcription factor gene mutations were linked to the formation of β amyloidosis and/or cornerstones β peptide aggregates and filaments (Banik et al., 2015). Botulism toxins, which are utilized to cause the buildup of senile lesions in patients with AD, are employed to induce physiological features. Conversely, PD animals are classified into neurotoxicity or genetically caused, and they incorporate cholinergic neurons loss in the globus pallidus (Blesa and Przedborski, 2014). In particular, neurotoxicity treatments produce sudden and strong cell death in the ventral striatum, as well as developmental delays and abnormal movements. Heritable traits rodents, on the other hand, show varied point, which is an important pathogenesis, as well as certain obvious manifestations such cellular disintegration and behavioral abnormalities. α-Synuclein is the main component that causes PD and AD with laws clumps (Henderson et al., 2019). The accumulation of α-synuclein, as well as the depletion of dopamine, happens in geographical cells called synapses throughout the brains. Specific genes can be used to illustrate gene variations, or antiviral translation can be used to cause chromosomes variations. The instances of frequently utilized laboratory animals for CNS disorder are given in Table 3.

**TABLE 3:** Neuroendocrine tumor, sickness of Alzheimer, and disease of Parkinson animal models are summarized.

Disease	Model	Characteristics	Reference	Plant name	Part used	Neuroprotective activity	Reference
GMB	Xenograft/HT1080 (human cell line)	IDH1 mutant (MGG152)	Tateishi et al. (2015)	*Fumaria indica* (Hausskn.) Pugsley	Leaf	Significant activity of ethanolic extract on rat cognitive dysfunctions. Potential antianxiety activity of leaf extract; preclinical study	(Singh et al., 2013a; Singh et al., 2013b)
	Xenograft/LNT-229 and LN-308 (human cell line)	IDH1 R132C mutant (HT1080)	Szabo et al. (2016)				
	Xenograft/BT111 (TIC), BT116 (TIC)	Unmethylated MGMT (BT111) and (BT116)	Sharpe et al. (2015)				
	Xenograft/U251 (human cell line)	N/A	Sharpe et al. (2015)	*Alhagipseud alhagi* (M. Bieb) Desv. ex B. Keller &Shap.	Whole plant	Traditionally used for neuroprotective disorders. Compounds having neuroprotective activity such as flavanone glycosides and alkaloids such as β-phenethylamine and tetrahydroisoquinoline have been reported	(Ghosal et al., 1974; Singh et al., 1999; Muhammad et al., 2015)
	Allograf GL261-Luc (mouse cell line)	N/A	Zeng et al. (2013)				
AD	PDAPP	Unspecified microgliosis and resistant microglia are both related to diseases	Cohen et al. (2013)	*Premna mucronata* Roxb	Whole plant	Luteolin and apigenin are reported, and they are reported to be neuroprotective	Dave et al. (2015)
	Tg2576	Plaques are related to neural stem cells that have been allocated a particular character	Elfenbein et al. (2007)				
	APP23	In specifically, the glial cell is associated in fibrils deposits, epidemic, and also cytosis	Karch and Goate, (2015)	*Semecarpus anacardium* L.f	Fruits	Stress-induced neuroprotective activity	Shukla et al. (2000)
	J20	Microgliosis and astrogliosis are two forms of engraftment that may occur in the brain and have negative consequences	(Hartley et al., 2015)	*Sida cordifolia* L	Whole plant	Ameliorative effect in parkinsonism	SJ and Kumar B, (2020)
PD	MPTP Neurotoxin: inhibition of complex I	No time passes during the process of accumulation, and cholinergic neurotoxicity occurs quickly and severely, resulting in a major motor deficit	Dauer and Przedborski, (2003)	*Tinospora cordifolia* (Thunb.) Miers	Stems	Suppresses neuro-inflammation in Parkinsonian Mouse Model; potential neuro-regenerative candidate against glutamate induced excitotoxicity: an *in vitro* perspective	(Sharma et al., 2018; Birla et al., 2019)
	6-OHDA Suppression of 6-OHDA suppression of enzyme I as well as antioxidant distress caused by neurotoxic	Heterogeneous sensory impairments, no synuclein aggregation, fast and severe cholinergic deterioration	Schober and research, (2004)	*Trichosanthes dioica* Roxb	Rhizome	Neuropharmacological properties of root	Bhattacharya and Haldar, (2013)
	UCH-L1 I93M mutation	In rodents, there were no synuclein aggregation, cholinergic neurotoxicity, or minor motor impairments	Setsuie et al. (2007)				

## Challenges of regular drug delivery to the CNS

**FIGURE 2:**
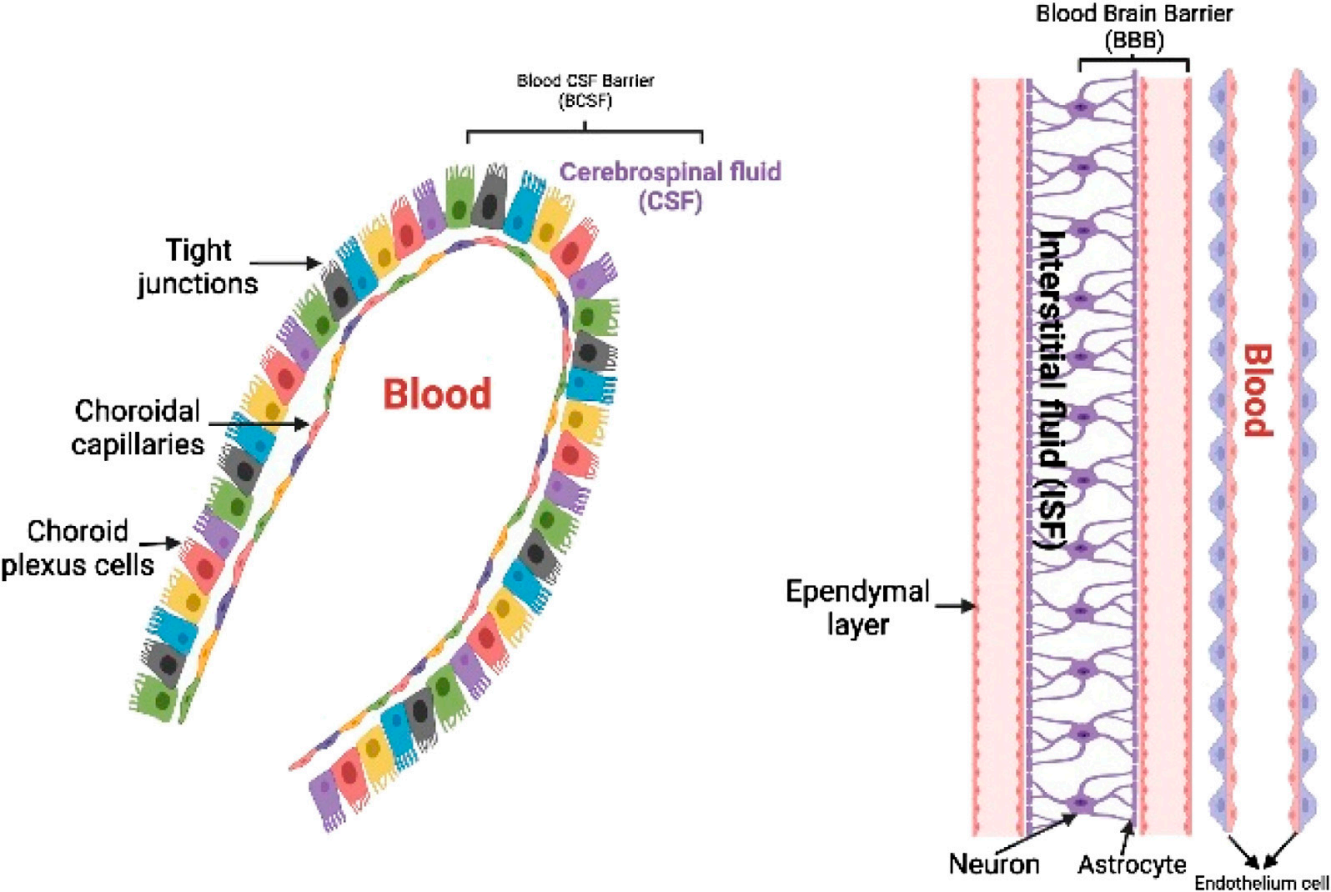
ynopsis of the blood–brain and blood–cerebrospinal fluid barriers. The blood–cerebrospinal fluid (CSF) barrier, which stops substances from traveling paracellularly into or out of the brain, is made up of tightly connected choroid plexus epithelial cells. Additional chemical barriers exist to stop substances from entering the central nervous system.

The medicine must be lipophilic in nature and have a low molecular mass (400–600 Daltons) in order to be successful in conventional chemotherapy (Pardridge, 1997). This transportation can be accomplished through percutaneous, passive, and other methods (Domınguez et al., 2013; Chauhan and Chauhan, 2015), although BBB provides for regulated entrance of prospective medicines (Crone, 1986; Jain, 2007; Stockwell et al., 2014; Yi et al., 2014). Slow drug activity, affiliation or transformation of the medication into non transporting forms, and poorer synaptic bioavailability are all main factors for pharmacological difficulties in the CNS (Tamai and Tsuji, 1996). Some enzymatic processes in the CNS also breakdown or keep dormant drugs in the brain that have a non-specific function (Crone, 1986). The thin blood vessel wall, which makes up the majority of the physiological barrier, is made of endothelial cells. Glial cells, specifically astrocytes, firmly encircle the capillary endothelial cells, with neurons nearby, and control BBB functions (Figure 2) (Zlokovic, 2008; Neuwelt et al., 2011; Yamazaki and Kanekiyo, 2017). The neurovascular connections are important for a healthy brain as well as the efficient administration of drugs (Woodworth et al., 2014; Wang et al., 2021). Other leukocytes located in the periphery, such as pericytes, oligodendrocytes, and extracellular matrix, among others, play an essential role in maintaining homeostasis in the brain as well as a healthy BBB. In addition, owing to the presence of tight junction proteins such as occludins, claudins, and tight junction’s adhesion molecule-A (Sweeney et al., 2019; Bhattacharya et al., 2020), large serum proteins and electrolytes are unable to pass across endothelial cells in a paracellular manner. Because of this, the BBB is responsible for establishing a distinct anatomical divide between the albuminal brain and the lumen of the blood vessel (Zeevi et al., 2010; Naik and Cucullo, 2012; Heidarzadeh et al., 2021). In addition to a physical barrier that is provided by the BBB, the endothelial cells of the brain possess an enzymatic activity that permits some compounds to be degraded before their passage over the BBB (Lorke et al., 2008; Upadhyay, 2014). Brain endothelial cells are known to express a number of important transport proteins, including organic anion carriers (OATs), P-glycoprotein/MDR1 (Pgp), multidrug resistance–associated protein 1 (MRPs), breast cancer–resistant protein (BCRP), and several other proteins (Englund, 2005; Löscher and Potschka, 2005; Sangha et al., 2022). These proteins control the pathway of several different xenobiotics and drugs. These proteins may inhibit the buildup of macromolecules in the brain by effluxing chemicals back into the blood supply. Nevertheless, the OATs have been discovered to be situated at almost all of the body’s barrier epithelia and the endothelium, and they have shown to play some roles in the management of intercellular mobility of several diverse organic anionic molecules across these epithelial barriers and between body fluid compartments. In addition, the endothelium has been found to be a localization site for the OATs as well (such as blood and the central nervous system, blood and urine, intestine and blood, blood and bile, blood and the placenta, and others). Regardless of the fact that the prototype members of this transporter family are capable of the bidirectional movement of substrates, the vast majority of organic anion transporters (OATs) are usually thought of as influx carriers, which aid in the passage of organic anions into the epithelial and endothelial cells. Because of these factors, the neuroprotective actions of the dysfunctional BBB make it visibly challenging to transfer medications to the brain, particularly in circumstances in which efflux carriers are hyperactive (Klaassen et al., 2013; Sanchez-Covarrubias et al., 2014; Saunders et al., 2016). Thus, it may be difficult for drugs to reach their target tissue or to concentrate enough in the brain, a problem that has been seen in a variety of conditions that affect the CNS to this day (Thorne and Frey, 2001; Doran et al., 2005; Bae and Park, 2011). Because of its significance in the medical community and the pharmaceutical business, the study of drug delivery has become an increasingly important topic of research over the course of the last two decades. This review delves into deeper information regarding recent studies and research initiatives that have proven inventive approaches to circumvent the BBB in medication delivery (Nance, 2019). These investigations and projects are discussed in this review in greater depth.

## Nanotechnology for drug delivery to the brain

Engineered adjustable devices with sizes in order of billionths of meters have been offered as an attractive tool, which is potentially able to answer the unmet problem of increasing transportation of drug over the BBB in recent years including the advent of nanomedicine (Re et al., 2012; Holmes, 2013). NP technology is quickly advancing across a variety of technologies. NPs are objects that range in size from 1 to 100 nm (Demetzos and Pippa, 2014) and function as a single unit in terms of transport and characteristics. The promise of NPs’ multi-functionality, along with their ability to deliver therapeutic payloads, including BBB impermeant medicines, is the primary factors of this enthusiasm. The use of NPs for drug delivery in the brain is demonstrated by the fact that adequate surface functionalization may increase either their targeting of the BBB or their enhancement of its crossing at the same time. When BBB impermeant pharmaceuticals are vehicled by NPs, their ability to cross the barrier is defined by the physicochemical and biomimetic properties of the NPs, rather than the chemical structure of the drug, which is impeded inside the NPs. The ability to confer on NPs’ features such as high chemical and biological stability, feasibility of incorporating both hydrophilic and hydrophobic pharmaceuticals, and the ability to be administered *via* a variety of routes (including oral, inhalational, and parenteral) (Petkar et al., 2011) makes them even more appealing for medical applications. Furthermore, NPs can be functionalized to target-specific tissues by covalently conjugating them to several ligands (such as antibodies, proteins, or aptamers). Multiple copies of a ligand can be added to NPs, also greatly increasing their binding affinity by multivalent functionalization (Montet et al., 2006), when developing NPs for therapeutic applications, by keeping in mind that their systemic delivery can cause significant changes. The nonspecific interaction between the shell of NPs, also several kinds of proteins circulating in the circulation, results in the adsorption of opsonin on the surface of proteins that generate the so-called corona. These proteins significantly alter the basic material qualities which govern NPs removal from circulation *via* the reticuloendothelial system, which is mostly found in the spleen, also in the liver. The most frequent methods for evading RES are constructing particles with a neutral surface charge, also covering them with different hydrophilic surfactants such as polysorbates and polyethylene glycol (PEG), and using small nanoparticles (e.g., 80 nm) (Provenzale and Silva, 2009). These “stealth” NPs are able to avoid the reticuloendothelial system which have a long circulation time including blood stability and can be functionalized to successfully target and penetrate the BBB (Gabathuler, 2010). Finally, NPs must be nontoxic to blood cells, healthy bystander cells, as well as biodegradable and biocompatible, also noninflammatory and nonimmunogenic (Andersen et al., 2010). It is also worth noting that designing NPs to improve drug delivery toward the brain does not always suggest that they can cross the BBB on their own. NPs could play this role in at least two ways, as expected: by boosting drug concentration inside BBB cells or at the luminal surface of BBB cells. This results in a local high concentration gradient between blood and the brain that is higher than that obtained following systemic delivery of the free drug. The gradient should therefore favor toward increased drug passive diffusion. This aim, for example, could be accomplished by creating NPs which are functionalized to target brain capillary endothelial cells. This property can be confirmed or disproven by their subsequent uptake from specific cells (Haque et al., 2012) and by transporting themselves and their drug cargo into the CNS. This task can be accomplished, for example, by allowing NPs to target brain capillary endothelial cells and after that transcellular transit through the BBB (Beg et al., 2019). The key aspects of NPs routinely used for medical purposes and already used or potential candidates for brain medication delivery will be outlined in the next part for the sake of completeness of this review.

### Inside the brain parenchyma: NPs diffusion

Although this feature is unlikely to be crucial when looking for delivery of therapeutic agents, it could be critical when looking for NPs to cross through the BBB, whereas the extracellular space (ECS) of the cerebral epithelium could hinder their dispersion or possibly prevent them from entering the brains. The opportunity to accomplish neural insertion with greater NPs is anticipated to help further homogeneousness which is longer lasting, as well as efficacious biomedical applications inside the nervous system. It also could be used to diagnose brain lesions, brain hemorrhage, neuronal injury, and other brain diseases where the BBB is undermined or municipal service methodologies which are conceivable (Masserini, 2013). It is now firmly understood that the ECS represents a volumetric proportion of between 15%, as well as 30% in healthy mature neural tissues, with a mean frequency of 20%, and this drops to 5% during worldwide hemorrhage (Syková and Nicholson, 2008; Wolak and Thorne, 2013). The exact magnitude of the intervals between the cell lines is seldom visible. Organic compounds such as inulin as well as sugar have a lower partition coefficient in the ECS than that in freshwater, implying that their transportation is restricted (Levin et al., 1970). 1) Cell membrane corridors; 2) entrapment of compounds in killed microspheres; 3) viscoelastic drag exacted by the biomolecules which assemble the collagen fibers or drag emerging from the wall surfaces of the networks while compounds are massive, such as in the situation of NPs; 4) temporary adhering to living cells or matrix proteins; and 5) nonspecific engagement with deleterious distribution on the surface of the NPs. Thorne and Nicholson (2006) offered an alternative type of framework to describe the greater compressibility empirically detected in the *in vivo* rat brain for larger molecules such as dextran and particularly huge synthesized nanoscale nanoparticles. It was hypothesized because nanocrystals with one hydraulic dimension of 35 nm were near to the median thickness of the ECS, as well as the predicted ECS breadth was 38–64 nm, regardless of whether a rectangular or cylindrical paradigm was used. Nance et al. (2012) claimed that NPs as massive as 114 nm in dimension disseminated inside the mammalian as well as rat brains only if they were thickly covered with PEG, in contrast to Thorne and Nicholson (2006). Researchers determined that actual normal brain cell ECS has some holes greater than 200 nm using those marginally sticky PEG-coated NPs. More than a fifth of all openings are less than 100 nm.

### Impact of protein corona

Nanomaterials’ capability to penetrate the brains may be determined by physical and chemical factors other than size. The creation of a “chromosphere” of macromolecules on the interfaces of nanomaterials, whose constitution varies relying on the pathway of access and the chemical and physical interface qualities of NPs, considerably complicates the scenario. The presence of a corona of macromolecules on the exterior of NPs could also influence whether they can pass from one capsule to the next and if these are efficiently picked up by cells (Pietroiusti et al., 2013). Depending on the location of distribution, nanomaterials are originally introduced to a physiological environment, such as bloodstream, in various situations where nanomaterials interface with living beings. For example, NPs implanted *via* IV infusion would indeed be introduced to bloodstream, which contains an abundance of enzymes and several other sophisticated bioactive molecules that compete with the nanomaterials’ membrane. According to prevailing thinking, the “naked” nanomaterials do not reside *in vivo* even though individuals which are instantaneously adapted by the adsorbate of hemoglobin in red blood cells with elevated affiliations for the surface of nanoparticles. This results in the formation of a too much or little covalently bonded stack (the as such tough corona) as well as a tenuously affiliated interactive surface (the so-called soft corona). Despite the reality that bodily liquids comprise millions of protein, it was discovered that ordinary tequilas have a restricted quantity and also different kinds of compounds were obtained from the bloodstream when this topic was researched (Mahon et al., 2012). It is also worth noting that perhaps the corona might play a significant role in those other negative consequences of NPs in biological processes, such as cytokine production and plasma coagulation, and is not always involved in intracellular ingestion. This should be noted that none of the *in vitro* studies looking into the mechanism of NPs transportation to the brains take into consideration the membrane functionalization of NPs within the bloodstream and whether it can influence BBB bridging. This is a subject that requires more research. Furthermore, after being ingested by endothelium BBB units, NPs may depart the brains wrapped in various macromolecules, also depending on whether they have completed endosomal escape, transcellular, or pinocytosis, and hence impose considerable impacts (e.g., cytotoxicity) on neurons. Moreover, that matter has still not been explored (Masserini, 2013).

### BBB changes in neurological diseases

The BBB’s unique pharmacological properties influence medication ductility and, for most situations, severely restrict the quantity of medications absorbed by the brains. It really should be noted since the physiology and structure of the BBB are modified in a variety of clinical states, and these alterations could have had an impact on physiologically circulating compounds, medicines, as well as NPs traversing the barriers. It also should be noted that these alterations are not uniform across all illnesses: variation is significant, making it difficult to anticipate the consequences of any given medicine. The BBB changes could affect the dosing, effectiveness, and adverse effects of regularly utilized medications in general (Masserini, 2013). The impact of hypoxic conditions on the barriers, for example, has been thoroughly studied. Oxygen starvation triggers a cascade of processes that enhance BBB vulnerability, perhaps due to limited connection breakdown and regulated by secondary messengers such cytokine and nitrous oxide (Yang and Rosenberg, 2011). Considering this scenario, biomolecules’ transit through the BBB is anticipated to rise. In laboratory animals of hypoxic conditions, nanocrystalline antidiuretic hormone has been shown to preserve neurons (Chen et al., 2012). Albumin has been demonstrated to penetrate target cell from the bloodstream in animals (Deng et al., 1995), suggesting that the BBB is similarly disrupted in septic encephalopathy. Although there is no indication of a significant rupture of the BBB in AD in individuals or experimental animals, the AD brains do require less sugar and oxygen. It is uncertain whether the reduction is due to a fault in the circulation through the membrane or a reduction in CNS demand; nonetheless, it is compatible with the AD microenvironment encouraging barriers and cellular discharges which are deleterious to cognitive health (Sharma et al., 2012).

### Size

One underappreciated question is when the diameter of NPs developed for CNS medication distribution creates a contribution. Various inquiries have been conducted in an endeavour to elucidate the situation. Sarin et al. (2008) discovered that NPs may pass through holes in the blood–brain tumor boundary of RG-2 aggressive glioblastoma after being systemically supplied with synthesized PAMAM microparticles with a diameter of about 12 nm. Sonavane et al. (2008) investigated the biodistribution of aqueous metal nanoparticles of various sizes (15, 50, 100, and 200 nm) administered intravenously to rodents. The number of NPs that penetrated the brains within 24 h was inversely related to their mass, with 15 nm gold NPs having a 500-fold amount than 100 nm NPs and 200 nm NPs having a relatively small quantity. Nevertheless, for 15 and 50 nm, the overall amount of currency, which really is proportional to the entire capacity of NPs penetrated to the burden of medication in the instance of drug-loaded NPs, was comparable, as well as approximately 30% less for 100 nm. Models are used to describe (Oberdörster et al., 2004) created crystalline nanoscale graphene nanoparticles with a diameter of roughly 36 nm that have been inhaled by animals. The researchers noted whether flying NPs of such magnitude can reach the CNS through the olfactory bulb from the olfactory mucosa. Emerging investigation uncovers whether the passage of NPs throughout the digestive tract has a significant impact on their future accessibility to the brains. Hillyer and Albrecht (2001) investigated the digestive assimilation and vasculature dissemination of magnetic dispersion of gold nanoparticle with diameters of 4, 10, 28, and 58 nm. The golden nanoparticles were administered orally, and golden concentrations in several tissues/organs, including the brain, were measured after 12 h. The overall quantity of metal effort to uphold in the brain was comparable for 4 nm and 58 nm particle diameter, according to the study. Schleh et al. (2012) looked into the effect of golden nanomaterials size and morphology charged on diffusion through gastrointestinal obstacles and accumulated in the brain following oral treatment.

## Nanomethods for delivering CNS drugs

Nanotechnology is a novel, effective, and cutting-edge method of delivering neurotherapeutics across the blood–brain barrier. Nano-medicines have shown considerable promise in CNS drug delivery over the last few decades due to their nanosized range, certain physicochemical features, and capacity to leverage surface-tailored biocompatible and biodegradable nanomaterials (Kaur et al., 2008). Site-specific transport of medicines including other chemicals across the BBB using nanotechnology might potentially be tailored, which can perform specific roles whenever needed. The medication, which is a component of the nanoengineered complex with other essential activities, is the pharmacologically active component to be given, for example, encapsulating the active drug, protecting it from enzymatic degradation, allowing it to release at certain pH, crossing the BBB, and targeting specific brain cells (Silva, 2008). Liposomes, PNPs, SLNs, micelles, dendrimers, and numerous other pharmacological nanocarriers have all been created (Rajadhyaksha et al., 2011; Wong et al., 2012; Ozkizilcik et al., 2017).

### Micelles

Micelles are vesicles made of amphiphilic surfactants (non-polymeric micelles) or amphiphilic copolymers (polymeric micelles), and they have lately attracted the attention of scientists because they may be used as a means of delivering drugs to the CNS (Aliabadi and Lavasanifar, 2006; Torchilin, 2007). Polymeric micelles have a longer duration of action and higher biodistribution than non-polymeric micelles (Ozkizilcik et al., 2017). They have a core-shell structural design with a size range of 10–100 nm that is made up of an outer hydrophilic environment primarily composed of polyethylene glycol (PEG) and as well as an inner hydrophobic core composed of molecules such as polycaprolactone, polypropylene glycols, phospholipids, and fatty acids, allowing hydrophobic drugs to be loaded (Torchilin, 2007). In an aquatic environment, the outer hydrophilic coating keeps micelles stable. In addition, it extends the time they spend circulating in the blood, shielding them from the reticuloendothelial system (RES) and increasing their accumulation in leaky vascular areas (Ozkizilcik et al., 2017). Because it may block drug efflux transporters, the pluronic copolymer class, commonly referred to as poloxamers, is of particular interest. Drug distribution to the CNS is made easier by the P-gp efflux carrier, which is widely expressed on the BBB (Park et al., 2008). In addition, they have been shown to increase drug solubility and stability in plasma as well as the distribution of low molecular mass drugs into the brain. Numerous efforts have been made to change micelles in a manner that enables a larger concentration of loaded medication to pass the BBB on the opposite side without much difficulty. One such modification is the binding of polyclonal antibodies to the receptor on the luminal side of the BBB against the brain-specific antigen, 2-glycoprotein, or insulin. When these modified micelles are administered intravenously to mice after being loaded with either a fluorescent dye or the neuroleptic drug haloperidol, both the fluorescent dye transport and the neuroleptic activity of the drug are significantly enhanced (Batrakova and Kabanov, 2008). Another variation of the micelle approach involves direct conjugation of the drug molecule and the targeting moiety to the amphiphilic area. For instance, we studied cyclo(Arg-Gly-Asp-d-Phe-Lys) paclitaxel conjugate–loaded micelles modified with transferrin and discovered better absorption by brain microvascular endothelial cells *in vitro* as well as prolonged retention in glioma tumors *in vivo*, both without considerable toxicity (Naqvi et al., 2020). The interaction of chitosan oleate self-assembled polymeric micelle–based nano-systems and polylactic–glycolic acid (PLGA) NPs coated with chitosan oleate (CS-OA), which may be conferred as a positive surface charge, with Caco-2 and Hela cells was investigated. According to data from cell line interactions, TGA studies, and influence the way, PLGA-CS-OA was shown to be more stable than polymeric micelles when it was packed with the lipophilic drug carrier resveratrol (Zhang et al., 2012).

### Liposomes

The first generation of novel colloidal nanocarriers, known as liposomes, were developed and tested as a medicine delivery system in the 1970s (Bawarski et al., 2008). These are tiny, spherical vesicles that include one or more phospholipid bilayers around a hydrophilic compartment in the core. This has morphological similarities with cell membranes, which are used to transfer medicines, proteins, and peptides (Martins et al., 2007; Wong et al., 2012; Ozkizilcik et al., 2017). The three varieties are classified according to their size and number of bilayers as small unilamellar (10–50 nm), gigantic unilamellar (50–1000 nm), and multilamellar (20–100 nm). In addition to hydrogen bonding between molecules, non-covalent interactions such as van der Wall forces also result from this. These buildings can be turned around (Ramos-Cabrer and Campos, 2013). Normal, unaltered liposomes circulate in the body for a brief period of time before being swiftly eliminated from the systemic circulation by RES cells. The creation of long-circulating and tailored liposomes has so been the subject of various efforts. It has been discovered that covering liposomes with polyethylene glycol (PEG) may successfully evade RES detection of nanocarrier systems (Doxil, 2012; Wong et al., 2012). The targeted delivery of PEG-modified liposomes to the brain may be improved with further modifications using ligands such monoclonal antibodies (mAbs) against glial fibrillary acidic proteins, transferrin receptors (TRs), or human insulin receptors (Pardridge, 1999; Miele et al., 2019). Because of receptor-mediated endocytosis, transferrin-conjugated liposomes have been shown to transfer payloads, such as 5-fluorouracil to the brain, more effectively (Shah et al., 2013). Prednisolone-loaded liposomes and mAbs that are recognized by cell surface receptors in the targeted tissue are combined to form immunoliposomes, which have been shown to have improved liposome dispersion inside the brain and to be highly effective against experimental autoimmune encephalomyelitis (Soni et al., 2008). It has been shown that immunoliposomes, which are nanocarrier systems for brain medicine administration, may also be employed in gene therapy by linking a TRsMAb-targeted liposome with a plasmid for tyrosine hydroxylase in the treatment of Parkinson’s disease in a rat model. Small interfering RNA (siRNA) targeting the epidermal growth factor receptor (EGFR) has also been delivered *via* this technique, leading to EGFR knockdown and improved survival in mice implanted intracranially with brain tumors (Schmidt et al., 2003; Zou et al., 2010). Another method of enhancing liposomes’ capacity to penetrate barriers and improve therapeutic efficacy is to modify them with cell penetrating peptides. For instance, doxorubicin-encapsulated liposomes may be used in combination with the specific ligand transferrin (T7) and non-specific cell penetrating peptide (TAT), which has been shown to have high availability across the BBB and specific cell targeting to brain glioma (Pardridge, 2007). When in contact with water, nimodipine PR liposomes form a liposomal structure. This has just been shown to increase the medication’s oral absorption (Zong et al., 2014).

### Polymeric nanoparticles

These polymeric nanoparticles are solid colloidal dispersions of biocompatible and biodegradable polymers with sizes ranging from 10 to 100 nm, such as PACA, PLA, PLGA, and natural proteins and polysaccharides (García, 2009). Polymeric nanoparticles can be used in a variety of applications, including biodegradation, biocompatibility, and biodegradation. Lipophilic medications are encapsulated in a thick polymer matrix core, while a hydrophilic corona provides steric stability to NPs. The NPs’ surface might be used to encapsulate, adsorb, or chemically bind the drug to be delivered (Parveen et al., 2012b). PEG and other hydrophilic polymers have been shown to increase the NP’s shelf-life in systemic circulation, which may be achieved by the addition of tissue-targeted polysaccharides or other hydrophilic polymers. When combined with traditional therapy, coating poly(n-butylcyanoacrilate) NPs with 1% polysorbate 80 (PS80) has been shown to enhance the concentration of rivastigmine or tacrine medication in the brain while decreasing hepatic or gastrointestinal adverse effects (Mohamed and van der Walle, 2008; Wilson et al., 2008). Dalargin nanoparticles coated with PS80 were reported to pass the blood–brain barrier and elicit antinociception upon oral distribution in another investigation (Das and Lin, 2005). It is possible that PS80-coated PACA nanoparticles injected intravenously may bind to ApoE and B in the blood, which can then be transcytosed across the BBB through low-density lipoprotein receptors (Kreuter, 2013). PLGA nanoparticles may transport medicine over the BBB without damaging it. C57/bl6 mice and hCMEC/D3 cells were used to study the effects of vanlafaxine-loaded PLGA nanoparticles on depression. P-gP pump efflux does not influence intranasal delivery of receptor-mediated PLGA nanoparticles, which leads to improved biodistribution in the brain and does not alter P-gP pump concentration in the basolateral side after 24 h through receptor-mediated endocytosis (Cayero-Otero et al., 2019).

## Drug delivery methods for the brain

The BBB operates as a microvascular endothelium connection, allowing important chemicals and ionic transfer to the brain (Tamai and Tsuji, 1996). The BBB is usually a major stumbling block for medicine administration systems. Water-soluble compounds have been found to migrate via particular carrier-mediated endocytic, transporters, and the extracellular channel. Dispersion and addition to introducing have been used to transfer liposomes compounds (Stenehjem et al., 2009). The following are examples of drug distribution pathways.

### Invasive approach

This mechanically violated approach breaks through the BBB and inserts the medicine straight into the brains. It necessitates a brain surgery for intracranial hemorrhage medication delivery and intracerebroventricular (ICV) injection (Stenehjem et al., 2009; Gabathuler, 2010). Collapsing the tight junction of endothelial causes BBB disturbance for drug diffusion (Inamura and Black, 1994; Stenehjem et al., 2009). Osmolarity disruption (Neuwelt et al., 1991; Rapoport and neurobiology, 2000) or destructive plasma dissolved substances can be used to deliver this (Aird, 1984; Erdlenbruch et al., 2003). Because the medicine is carried in the periphery circulating blood rather than the different tissues, ICV delivery of drugs is regarded a poor technique (Lee et al., 2011). Instead of advancing high molecular weight drug absorption, ICV was confined to confined drug dealing and lack of intended CNS action following straight prescribed medication owing to excessive intraocular pressure (Soni et al., 2016a).

### Pharmacological approach

The spontaneous inactive passage of medicines *via* the blood–brain barrier is the basis for this empirical methodology (Stenehjem et al., 2009; Gabathuler, 2010). Because of their tiny molecular size, poor hydrophobic interactions capability, and solubility in water, these compounds can traverse the blood–brain barrier without assistance (Hsiung et al., 2003). Physiological changes, such as a decline in the amount of polar functional groups, are also part of this strategy, which improves medication transport across the BBB (Pardridge, 1998). If the solubilization of the changed particles increases by several folds, it may operate as a P-glycoprotein outflow pump (Stenehjem et al., 2009).

### Physiological approach

Drug transport to the CNS *via* receptors and carriers is deemed a cutting-edge pharmacological approach (Stenehjem et al., 2009; Gabathuler, 2010). The BBB is frequently seen with associated protein and insulin channels (Gabathuler, 2010). As a result, the medicine may convey pharmaceuticals to the specified cerebral location if it interacts with the ligands of these channels. The medicine must be capable of mimicking the indigenous carrying substrates when using transporter-mediated distribution (Allen and Cullis, 2013). However, the pharmacokinetics and interaction capability of the carrier molecules constrain the administration of CNS drugs *via* a pharmacological methodology.

## Treatment of CNS disorders with nanocarriers and BBB-bypassing techniques

The presence of the tightly selective BBB is one of the greatest roadblocks in the development of successful CNS therapies. As a result, the use of various nanocarriers in combination with various administration routes may be of tremendous interest in bypassing the BBB.

### Parenteral drug delivery

**FIGURE 3:**
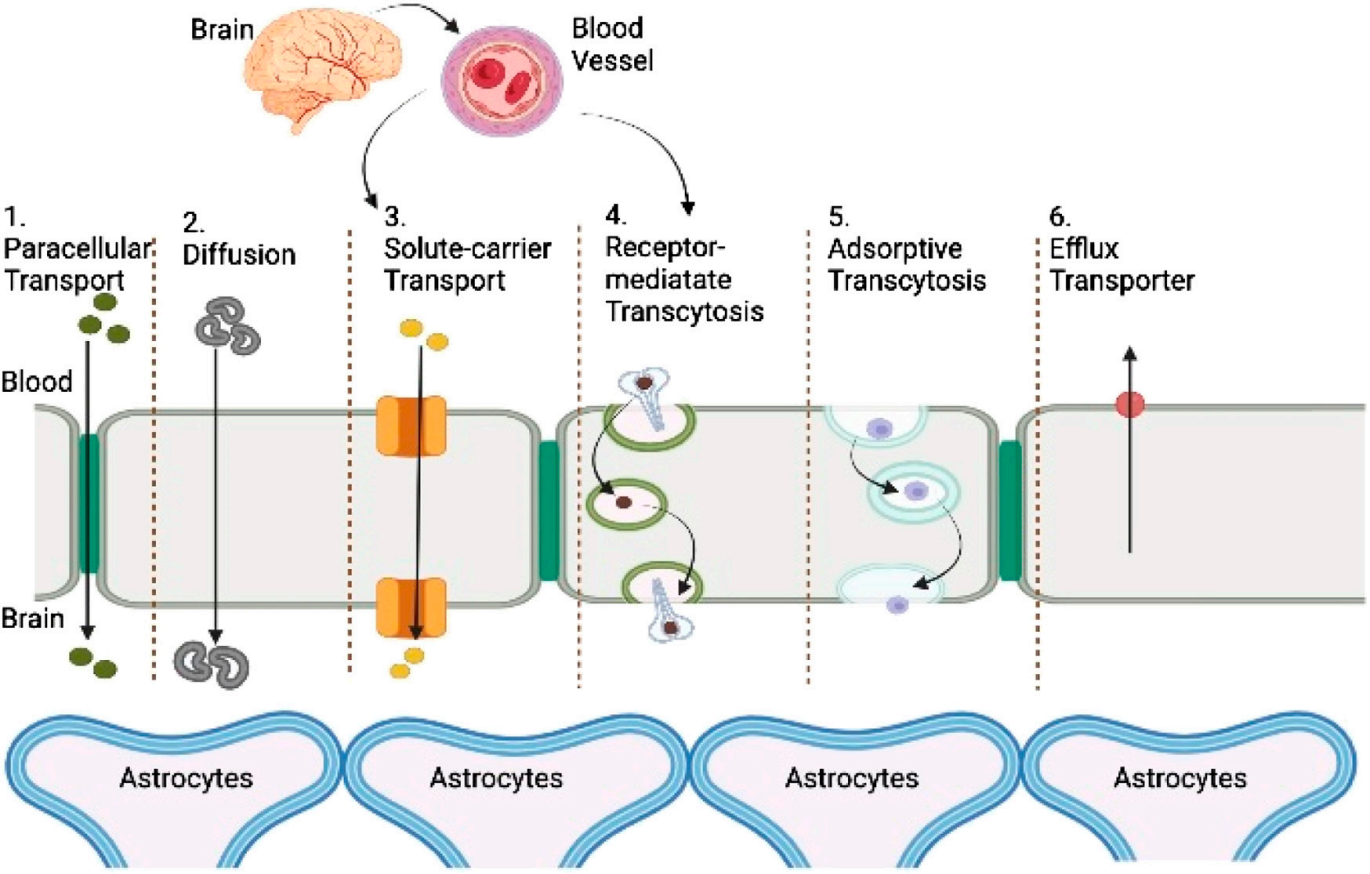
Representation of transport routes across the BBB. Passive transport: ionization of the medication, molecular weight, lipophilicity, and protein binding are the key elements that affect passive transfer through the BBB. When acidic substances are ionized, their transport through the BBB is reduced.

Since IV injections are less invasive and allow precise control over bioavailability, thorough dosages of frequently toxic drugs, and continuous administration of several-hour injections, obtaining a relatively better drug concentration within the duration of treatment is the most effective mode of injecting drugs for CNS afflictions. Medicines given intravenously, engaging other organs or cell, should either have a greater possibility to passively disperse through the BBB (such as the two very hydrophobic medication including carmustine and temozolomide) or employ one of the aided transcytosis pathways, as shown graphically in Figure 2. Exogenous chemicals may utilize the BBB’s most common facilitators and transporters to access the brain barrier, according to an assessment by Griffith et al. (2020) and Mulvihill et al. (2020). Nanotherapies may be implicated in a wide range of transcytosis pathways because of their heterogeneous physicochemical character (Figure 3). Furthermore, to stimulate a sufficient brain translocation, their tridimensional properties almost always necessitate the appearance of a certain specific side on their surface. In addition to the widely utilized intravenous delivery, which requires a special design of nanoparticles to a specified site and circumvent the BBB, direct intracerebral drug infusion is another viable but quite invasive technique of administration. This surgically aided method of administration can be accomplished either by stereotactic injection in the diseased area (i.e., the intraparenchymal route) to build local depots or by intraventricular administration (Griffith et al., 2020). Because of their high danger, these invasive administration methods are only used in life-threatening situations and do not allow for repeated injections (Yi et al., 2014).

### Intranasal drug delivery

Because it permits the formulation to circumvent the BBB, for brain targeting, intranasal, or nose-to-brain pharmaceutical delivery is a highly fascinating form of distribution that has just developed. The respiratory and olfactory parts of the nose can both be used to carry out this operation. Millions of olfactory neurons reside in the nasal epithelium, allowing substances to be transported directly to the brain *via* transcellular diffusion *via* the olfactory bulb (Griffith et al., 2020; Mulvihill et al., 2020). The second channel involves the respiratory epithelium, which allows drugs to directly reach brain tissue *via* the trigeminal nerve (Griffith et al., 2020). As recently reviewed by Yi et al. (2014), the intranasal route has been successfully tested in a range of neuroinflammation-related illnesses.

### Intracarotid infusion

The intracarotid infusion technique involves injecting a medicine or fluid into the carotid artery, which is the main blood vessel that connects the heart and the brain (Joshi et al., 2008). Intracarotid drug delivery can provide extremely high local concentrations of the unique pharmacological agent in a matter of seconds, facilitating regional delivery. This technique has long been overlooked in the development of medications for CNS illnesses, and the kinetics of such a route of administration is still unknown. However, a few studies have indicated that it can be an effective delivery method, particularly for the treatment of brain tumors (Inamura et al., 1994).

### Transmucosal drug delivery

Transmucosal drug administration is a novel and popular approach of direct CNS drug injecting that uses heterotopic mucosal grafts to circumvent first-pass metabolism and enables for fast drug uptake into the systemic circulation (Abhang et al., 2014; Pandey et al., 2016; Dong, 2018). This strategy is based on “cohesion”, or attachment to the human mucosa, which is extremely close to the blood circulation and may even transfer high molecular weight and polar substances to the CNS. This approach may be adapted to accommodate items delivered by nasal and oral routes by utilizing of droplets, creams, pills, or even suppositories as delivery systems (Bleier et al., 2013).

### BBB disruption caused by pharmacological or physical activity

To allow the brief passage of therapeutic substances such as nanocarriers, the BBB’s physical integrity can be partially and temporarily disturbed. These bioactive disturbances can be achieved by “physically” broadening and opening tight junctions, such as by utilizing an external ultrasonic (US) source to drive an electromagnetic differential to propel nanomaterials throughout the BBB or microscopic pores throughout the BBB (Teixeira et al., 2020): the nanomaterials can be osmotic (sorbitol, sugar, lactose moieties, ammonia, or glycerin) or form effective (cholecystokinin, antihistamine, tryptophan, and glutamine) or pro-inflammatory (particularly, prostaglandins and interleukins) or biologically active substances or tumor necrosis factor (Chenthamara et al., 2019; Xie et al., 2019).

### The ECM as a barrier to nanopharmacology

Because of the fact that it slows down the diffusion of macromolecules, the extracellular matrix (ECM) might be considered a barrier to the administration of nanomedicines. Hyaluronidase, an enzyme that breaks down HA, was injected before the NPs; this resulted in a more even dispersion of the NPs throughout the tissues (Lee et al., 2021; Liu et al., 2022). Nevertheless, improved NP distribution is attainable even without altering the ECM design. Therefore, paclitaxel (PTX)-loaded PEG–PLGA block copolymer NPs, which have a mean particle size of 70 nm, are able to diffuse through brain tissue one hundred times more quickly than identical particles that do not have PEG coverings. In accordance with this, the local administration of strongly PEGylated PTX-loaded nanoparticles dramatically slowed down the development of 9L liposarcoma as compared to PTX-loaded PLGA nanoparticles or unencapsulated PTX (Palei et al., 2018). Despite this, the diffusion of 100-nm PEG-modified particles was eight times slower in 9L liposarcomas compared to normal brain tissue. This was because the tumor tissue had a larger cell density and more extensive collagen content than normal brain tissue (Soni et al., 2016b; Henrich-Noack et al., 2019). These findings demonstrate that a combination of a vector targeting the ECM with a dense layer of PEG on the surface of NPs could improve the efficacy of the NPs by reducing their high emphasis with brain tissue and enhancing their percolation into the tumor (LeClaire, 2021). The perivascular space and lymphatic systems are also important factors that should be taken into consideration in relation to the diffusion of NPs in brain tissue (Khawar et al., 2015). It has been proven that the perivascular space offers a favorable permeable channel for the rapid convective transport of large NPs across tissue. This is true in particular for the transportation of bigger particles, the passage of which is impeded by the ECM (Voigt et al., 2014; Witten and Ribbeck, 2017). In point of fact, this might be the mechanism that lies behind the supposed presence of channels inside the ECM that make it easier for NPs to move about.

### Nanosystems’ possible side effects on the ECM

In addition to this, we need to take into account the possible impacts that NP have on the structure of the ECM. Therefore, silver nanoparticles may activate an inflammatory signaling pathway, leading to an upregulation of matrix metalloproteinases (MMPs) and an induction of ECM breakdown (Raja et al., 2020). In addition, it has been shown that the cytotoxicity of multiwalled carbon nanotubes may be altered *in vitro* depending on the kind of endothelial cells they are exposed to.

### Brain tumor nanotheranostics

T1-weighted MRI is used to guide the treatment of malignant brain tumors using image-guided therapy. Gadolinium chelates are often employed in contrast-enhanced T1-weighted MRI imaging of malignant brain tumors to differentiate between white and gray tissue. Because the BBB is disrupted in the tumor location in order to accurately define the tumor borders, these contrast chemicals do not flow into healthy brain areas. In order to improve the contrast agent’s sensitivity and efficacy, scientists are working on developing customized gadolinium-loaded nanocarriers (Fink et al., 2015; Aparicio-Blanco and Torres-Suárez, 2018). Thus, amphiphilic compounds containing gadolinium in the chelated form as a T1-weighted MRI contrast agent for brain nanotheranostics have been created. Currently, radiation is the usual treatment for malignant brain tumors, and the T1-weighted MRI imaging function has been used for theragnostic reasons (Gløgård et al., 2000; Richard et al., 2008). It is hypothesized that gold nanoparticles coated with gadolinium chelates (zeta potential: −30 mV) can absorb high energy ionizing radiation to cause thermal ablation of tumors, and thus act as a radio-sensitizing agent, in rats orthotopically implanted with a 9L liposarcoma, which can be used for image-guided radiotherapy (Simonet, 2018). It is possible to monitor the distribution of gold nanoparticles after intravenous injection using T1-weighted MRI since gadolinium had been added to the solution (Jung et al., 2012). This helped establish the best time to administer the X-rays. Comparing treated to untreated rats, the median survival period following tumor implantation was days, a 473% improvement over the non-radiosensitive nanotheranostics radiosensitizing treatment (median survival time: 2.5 days after tumor implantation). X-rays penetrate deeper into the tissue than visible light, making this thermal treatment a viable option for brain tumor theragnostic. Gadolinium chelate (particle size: 2 nm)–doped polyciliate nanoparticles were then used to investigate gadolinium’s radiosensitizing potential. After utilizing MRI-guided microbeam radiotherapy to target the tumor while sparing healthy tissue, the researchers were able to achieve a five-fold increase in median survival time, and half of all liposarcoma-bearing rats survived 100 days after tumor implantation using gadolinium-rich microbeam radiotherapy (Sancey et al., 2014; Bechet et al., 2015). Bechet et al. developed 3-nm-sized silica-based nanoparticles (zeta potential: 22 mV) functionalized with an anti-angiogenic heptapeptide (ATWLPPR) that targets neuropilin-1, an endothelial receptor found only on malignant brain tumors. This was done to improve the distribution of gadolinium-containing nanotheranostics to malignant brain tumors. MRI contrast agent and photosensitizer for PDT of brain malignancies guided by interventional MRI were contained in these particles. Using MRI guidance, the optical fiber was stereotactically implanted for PDT in rats with the orthotopic glioma model U87 after intravenous injection of neo vasculature-focused nanocarriers (Kopelman et al., 2005). Significantly reduced intratumorally blood perfusion in rats treated with targeted nanotheranostics was seen in this study (80%). A histological study of brain slices after MRI-guided PDT with specific carriers showed vascular damage and swelling (Hirschberg et al., 2008).

### T2-weighted MRI

Because of the fact that they have a high magnetic susceptibility, superparamagnetic iron oxide nanoparticles are often utilized as T2 agents to produce contrast enhancement for the purpose of identifying watery tissue (Qiao et al., 2009; Yang et al., 2009). The degree of tissue contrast enhancement is dependent on vascular extravasation, much as it is with T1 agents. As a direct result of this, brain-targeted iron oxide nanoparticles have been produced for the purposes of glioma therapeutic research. Since superparamagnetic iron oxide nanoparticles are already in use as MRI contrast agents, and since they can be easily upgraded to nanotheranostics by encapsulating various therapeutic agents, there is a significant possibility that they will be used in the clinical translation of brain diagnostics (Dehaini et al., 2016; Ag Seleci et al., 2021). For the purposes of theragnosis, various anticancer agents (drug substances, genes, and antibodies) or photosensitizers can be loaded into iron oxide nanoparticles. This allows for the possibility of combining MRI with chemotherapy or photodynamic therapy for the purpose of providing localized treatment (Aljiffry et al., 2009; Barreto et al., 2011). In addition, iron oxide nanoparticles themselves simultaneously exhibit therapeutic properties. These properties include the generation of heat under alternating magnetic fields, which can be used for the thermal ablation of tumors (Estelrich and Busquets, 2018; Tong et al., 2019). As a result, iron oxide nanoparticles are capable of functioning independently as brain nanotheranostics (Nelson et al., 2020; Lorkowski et al., 2021).

### Other imaging techniques

Although alternative scanners have a great potential for use in brain imaging, their use as diagnostic tools for brain disorders have not yet been investigated (De Bie et al., 2010; Pereira et al., 2016). Unfortunately, the majority of research that describe the performance of brain diagnostic nanomedicines *in vivo* examine either the therapeutic or imaging function, but seldom examine both of these functions at the same time. Imaging and therapeutic chemicals are often loaded independently onto a particular nanocarrier so that each may be evaluated independently for their biodistribution and effectiveness (Duncan and Gaspar, 2011; Chen et al., 2013). In these independent investigations, a larger armamentarium of imaging methods (radionuclide-based imaging and optical imaging, respectively) and nanocarrier architectures (liposomes, polymer nanosphere, and solid lipid nanoparticles) have been employed (Sanson et al., 2011; Butler et al., 2016; Yao et al., 2016; Houston et al., 2020). The development of theragnostic nanomedicines with multiple imaging functions is an additional promising future area of research. These nanomedicines would improve the sensitivity of monitoring the response of tumors to treatment, as well as guide stimulus-responsive therapies and surgical resection of malignant brain tumors (Nurhidayah et al., 2019; Das et al., 2020). Even though various nanocarriers with numerous imaging functionalities have previously been produced and tested in orthotopic models of malignant brain tumors, the coupling of these nanocarriers with a treatment method has not yet been explored *in vivo* (Baumann et al., 2013; Dixit et al., 2015; Aparicio-Blanco and Torres-Suárez, 2018).

### The effect of nanobiotechnology in nanomedicines to treat CNS diseases

The term “nanotechnology” originates from the Greek word “nano,” which translates to “dwarf”. Nanotechnology refers to the production and utilization of materials, equipment, and frameworks through the control of matter on the nanometer-length scale, or more specifically, at the level of atoms, molecules, and supramolecular structures (Fulekar, 2010; Ramanathan, 2012). It is the common term for the construction and utilization of multiple functions with at least one characteristic dimension measured in the nanometer scale. A nanometer (nm) is equal to one billionth of a meter (10–9 m), so this term refers to the construction and utilization of structures with at least one characteristic dimension evaluated in the nanometer scale (Nasrollahzadeh et al., 2019). This is about the same as having four times the diameter of a single atom. DNA molecules have a width of around 2.5 nm, whereas protein molecules range in size from 1 to 20 nm (Heng et al., 2006). It was inevitable that nanotechnology would be used for biotechnology, which gave origin to the name “nanobiotechnology”. Given the intrinsic nanoscale functional components of live cells, it was unavoidable that nanotechnology would be applied to biotechnology (Ball, 2004; Boisseau et al., 2007). The use of nanobiotechnology to medical practice is known as nanomedicine. The field of healthcare is already beginning to feel the effects of nanobiotechnology’s impact (Jain, 2008; Kumar et al., 2022). Over the course of the previous half century, early ideas about nanotechnology have given rise to a wide variety of technologies, and there are even a few treatments that are based on nanotechnology that are now on the market (Ratner and Ratner, 2003; Lemley, 2005). The phrase “nano bio-pharmaceuticals” may refer to a wide variety of applications within the pharmaceutical business. Some examples of these applications include drug discovery and drug delivery (Invernizzi, 2010). The development of the microscope ushered in a new era in medical practice by making it possible to identify microscopic organisms and investigate the histology of illness. Microsurgery was a significant improvement over crude microsurgery and opened the door to the possibility of surgeries that either had not been performed in the past or had a high mortality and morbidity rate. A comparable influence will be exerted on medical practice and surgical procedures by the advent of nanotechnologies, which will make it possible to see beyond the microscale (Morrow et al., 2007; Martel, 2016). This is due to the fact that both physiological and pathological processes that take place at the cellular level take place on a nanoscale. The incorporation of recent developments in genomes and proteomics into nanomedicine, which paves the way for the creation of customized medicine, is another way in which we may think of nanomedicine as an improvement on molecular medicine (Stratakis et al., 2009; Wei et al., 2017). A discussion on the connections between nanobiotechnology, nanomedicine, and other technological fields was carried out (Riehemann et al., 2009; Haseeb et al., 2020). This diagram provides an overview of how nanobiotechnology will influence the growth of nanomedicine both directly and indirectly *via* the enhancement of other fields, such as the distribution of nanomedicines and molecular diagnostics. Parallel to the development of nanomedicine, personalized medicine is made easier by the same technology that underpins nanomedicine.

## Neuro-inflammatory cell targeting with nanoparticles and modules

### Cell membrane coating strategies

Greater blood flow lengths and focusing capabilities have subsequently been demonstrated using biological membranes fragmentation as a nanomaterial interface covering element. Cellular membrane coating approaches offer immunological masquerade, lower neutrophil assimilation, and an appealing alternative for targeted non-immune cellular components including glial as well as synapses. Synthesis of cells plasma nanostructures (such as cellular barrier nanocrystals) and nanomaterials embellishment with biological membranes segments (membrane-coated nanomaterials or harnesses) are two innovative methods that permit development of novel therapeutic techniques for several diseases (Luk and Zhang, 2015). Cell-coated nanoparticles provide another option for avoiding immune detection, increasing prolonged circulation, and delivering targeted drug delivery. Various sorts of membrane surface could be employed for a range of specialized biomedical techniques without demanding substantial physiological modifications, which is clinically meaningful and can help the Food and Drug Administration to approve the product faster. Rapid innovations in the production of fibroblast nanomaterials that can discharge drugs for longer periods of time in responsiveness to the surroundings are paving the way for more complex treatment approaches, which could lead to distinct benefits for CNS drug delivery (Hu et al., 2011; Luk and Zhang, 2015; Liu et al., 2019).

### Neuro-inflammatory treatment using stimuli-responsive nanoparticles

A current emphasis of investigation has been the production of nanostructures which exhibit medicinal benefits in accordance to biological responses or application of an extrinsic stimulus. These materials, also known as stimuli-responsive materials, are designed to replicate the behavior of living beings. Formulating nanocarriers that release medications selectively at the target site in response to preset illness signals could revolutionize the development of successful neuroinflammation therapeutics (d'Arcy and Tirelli, 2014).

## Neurological disorders and diseases tested by nanomedicines

### Nanomedicine in stroke

Understanding the pathophysiology of ischemic stroke, as well as how nanoparticles engage with ischemic cells, is essential. The basic issue of how nanoparticles carry medicines over the BBB is still unknown, but new breakthroughs in nanoparticle design and manufacture, as well as innovative *in vivo* imaging tools (such as intravital microscopy) may be helpful. The patients with ischemic stroke will benefit from the development of new medicines and innovative nanoparticle-based drug delivery approach (Dong et al., 2020).

### Traumatic brain injury and nanotechnology

Traumatic brain injuries (TBIs) are injuries to the cerebrum tissue that cause temporary or permanent impairment of brain functions. TBIs cause 135,000 deaths and cases of lifelong disability in the United States each year. Acute respiratory distress syndrome (ARDS) is a life-threatening lung illness characterized by dyspnea, acute hypoxemia, reduced lung compliance, and diffused bilateral pulmonary infiltrates, among other symptoms. Patients with ARDS are severely unwell or hospitalized as a result of serious injuries, one of which is a TBI. Researchers have been looking for biomarkers in various biofluids for brain damage and its consequences. TBI biomarkers in urine xiv and serum that have been clinically validated still have low sensitivity and specificity (Vuong, 2018).

### Application nanomedicine in the treatment of brain tumors

**FIGURE 4:**
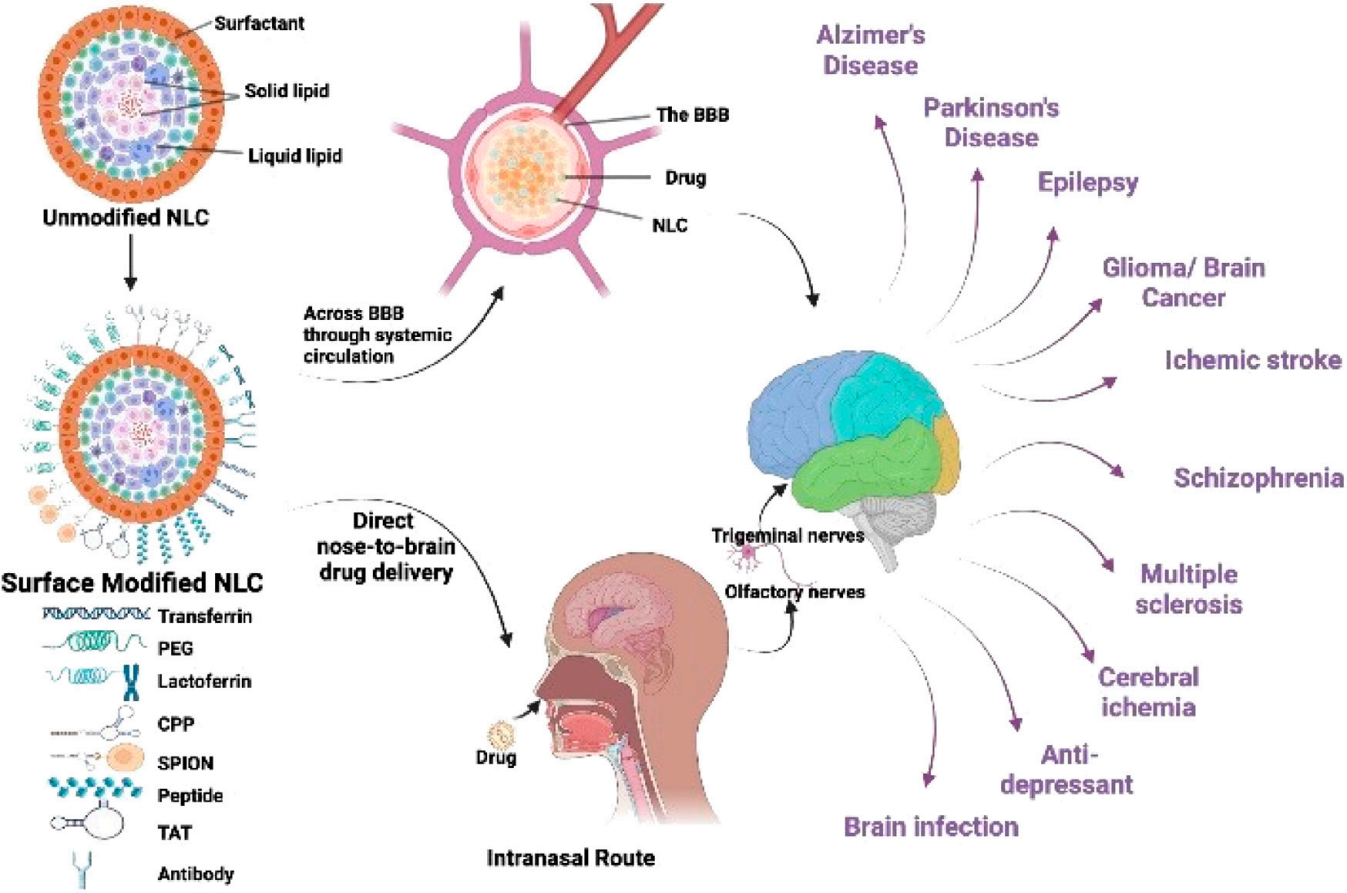
Strategies and advancements in the fabrication of nano lipid carriers for brain targeting.

Invasive brain CNS tumors account for approximately about 2% of all cancers. These are, nevertheless, typically associated with significant incidence and fatality percentages. Although recent standard-of-care interventions such as operation, radiotherapy, and chemotherapeutic are already present, there has yet to be curative or non-toxic therapies for aggressive CNS tumors. Nanoscience has the potential to transform this situation. It offers new promise in the identification and treatment of patients. This new technique could establish a framework for combining diagnoses, therapies, and transport to the cancer, as well as ongoing performance surveillance by creating and producing materials employing atomic and molecular elements. This study examines current advances in cancer nanotechnology, with a focus on nanoparticle systems, which are significant tools for improving medication delivery in brain tumors (Figure 4) (Invernici et al., 2011).

### Alzheimer’s disease

Since the discovery and definition of AD, the most common dementing dementia in the world, a century has passed in research. However, there are currently no definitive diagnostic procedures or viable treatments for AD. Furthermore, the currently available diagnostic methods are insufficient for early detection of AD so that preventive measures can be taken. Nanotechnology, a new field that has just emerged, has promised new approaches to address some of the issues associated with AD. Nanotechnology is the process of designing and fabricating nanoscale (1–100 nm) structures by manipulating the positioning and/or self-assembly of atoms and molecules in a controlled manner. In this study, we discuss the benefits of nanotechnology in AD detection and treatment research. They include the possibility for a greater knowledge of the molecular pathways that underlie AD, early detection of the disease, and successful therapy. The contributions of atomic force microscopy, single molecule fluorescence microscopy, and nano SIMS microscopy for AD research are discussed. In addition, the bio-barcode test, localized surface plasmon resonance nanosensor, quantum dot, and nanomechanical cantilever arrays, which were newly proposed uses of nanotechnology for the early diagnosis of AD, are examined. Nanotechnology applications in AD therapy are reviewed and studied, including neuroprotection toward oxidative stress and anti-amyloid therapies, neurodegeneration, and drug delivery across the BBB. All of these applications have the potential to improve treatment for AD and other NDs (Nazem and Mansoori, 2008).

### Parkinson’s disease

Screening imaging tools are being established and treatment instruments will rely heavily on nanotechnology. Nanostructures make use of manufactured nanomaterials with the tightest organizational structure on the nanoscale level in at least one parameter. Modification of certain substance qualities can lead to novel bioactive components. Nanomaterials could be utilized to create technologies which restrict as well as repair neurobiological disorder processes, cultivate as well as encourage efficient neuronal replacement, provide neuroprotective effects, and make drug and small molecule administration across the BBB easier. All of these are relevant for improving existing PD (Torres-Ortega et al., 2019).

### Spinal cord injury and nanotechnology in regenerative medicine

Because of the significant nerve damage, spinal cord injury (SCI) can result in the loss of perceptual and athletic function. Yet, pieces of evidence describing the exact pathogenic pathways in SCI remain ambiguous. The necessity for systemic distribution, which has a major negative effect on the patient, makes it difficult for pharmaceutical treatment to adequately relieve SCI impairments. As a result, developing SCI treatment options is both necessary and helpful. Nanopharmaceutical-based regenerative medicine will provide enormous potential space for clinical medicine as a result of the application of nanotechnology in pharmaceutical research. These nano pharmaceuticals (i.e., nanocrystalline medications and nanocarrier drugs) are made with various materials or bioactive compounds to increase therapeutic efficacy, lessen adverse effects, and distribute drugs quietly, among other things. Drug regulatory agencies are currently approving a growing number of nanopharmaceutical items, prompting more academics to focus on prospective SCI therapy options (Zhao et al., 2021).

### Nano-formulation approaches

For an influential drug delivery strategy in CNS therapy, nanoparticles affect the pharmacokinetics of drug and boost drug loading capacity (Khanbabaie and Jahanshahi, 2012). Before being loaded into various nanomaterial-based vehicles and delivered to the brain, drugs must be chemically changed (C Dinda and Pattnaik, 2013). It was also able to reach the brain *via* transcytosis through the BBB (Stenehjem et al., 2009). Nanobiotechnology has made significant progress in the realm of medicine delivery. We reviewed the features, nanotechnology-based medication delivery, and drug release mechanism using a few examples of patent nanomedicine (Table 4) (Haque et al., 2011).

**TABLE 4:** Treatment of neurodegenerative disorders used various potential nanostructures.

Neuro-degenerated disease	Nanomaterial	Outcome	References
AD	AGulX NPs	Enhancing the susceptibility of MRI, penetrating the BBB with minimal toxicity, addressing amyloid accumulations in the brain, and enabling excellent viewing of amyloid plaques without causing negative impacts	Pansieri et al. (2018)
	Boron-dipyrromethene biosensor	Might be utilized to analyze the accumulation of Aβ A in Alzheimer's disease and identify its self-assembly	Quan et al. (2019)
PD	Micelles	Intravenous delivery/efficient drug delivery	Brynskikh et al. (2010)
	Rasagiline (Double emulsion/solvent evaporation)	Significantly affect MAO-B enzyme protect your neurons from the oxidative stress	Myers, (2004)
MS	Quantum dot complexed with MMP-9 siRNA and MMP-1–loaded PLGA nanoparticles	Interpretation of MMP9 was significantly suppressed in the brain, as well as in microvascular endothelial cells and leukocytes. inhibition	Singh et al. (2017)
HD	Peptides QBP1, NT17, and PGQ9P 2 nanoprecipitation	Significantly affect mHtt clumping bring back the functioning of the motor systems	Joshi et al. (2018)

## Nanoformulations of natural compounds and herbal medications

### Polymeric nanoparticles, nanocapsules, and nanospheres

**FIGURE 5:**
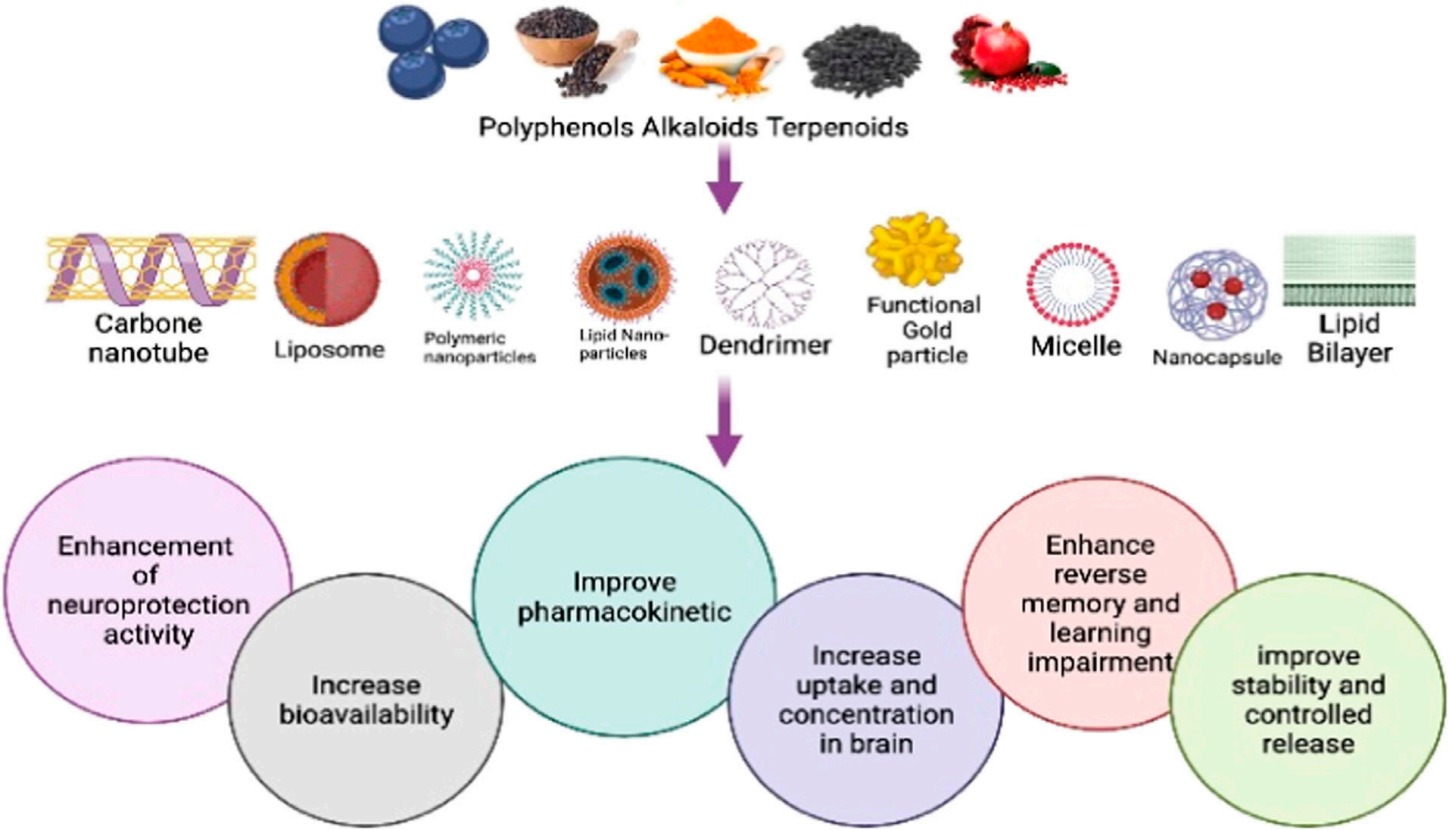
Nano-formulations are used to increase the potency of natural compounds.

Drug-loading capabilities of polymers nanostructures allow the device to safeguard and maintain the included medication toward breakdown. As a result, there is a greater possibility of drug incorporation and cerebral accessibility. They can elude neutrophils because of their stable architectures and distinctive characteristics, which facilitates the drug transport to the CNS. Although nano emulsions are created by a uniform thin wrapper enclosing a hydrocarbon chamber, nanomaterials are a robust elastomeric matrix created using micro-emulsion polymer (Figure 5) (Castro et al., 2022; Romero-Ben et al., 2025).

### Polymeric nanogels and nanosuspensions

Extremely covalently bonded submicron gelatin processes made of charge density, or standard quantity polymers are referred to as nanoemulsion. The hydrogels range from 20 to 200 nm range in size. The capability of this mechanism to load drugs is between 40% and 60%. Recent research studies revealed that polynucleotide consumption by hepatic and the spleen might be reduced and increased by hydrogels frameworks. Crystal dosage form stabilized by combinations of triglycerides or polymeric detergents make up matrix tablets nano emulsions. The ease of usage and impressive drug unloading and distribution capabilities of nano emulsion are only a few of their many benefits (Modi et al., 2010).

### Carbon nanotubes and nanofibers

Mesoporous silicon nanomaterials, CNTs, stacked dual ions in aqueous solution, ferromagnetic nanoclusters, as well as calcium phosphate nanostructures are a few examples of matrix composite targeted distribution that have found curative use in a variety of illnesses, including NDs. While improving drug concentration, penetration, retention impact, durability, and accessibility to desired places, properties of nano complexes can withstand extended blood vessels. This nanotechnology could also alter how a drug is released and make it easier to see and analyze a medication’s action. In addition, the CNTs are a remarkable find for nanopharmacology due to their flexibility to a variety of stimuli, including heat, pH, compounds, pressures, and magneto electric forces. Many of the most notable methods for neuropsychiatric applicability is the use of carbon-based nanomaterials, such as CNTs. Carbon alloying elements with tubular nanomaterials are known as CNTs. To improve their electrical currents, CNTs are currently the subject of intensive research. Direct electrical stimulation is one of the most efficient methods for treating many psychological and neurological conditions, particularly PD. When these activating conductors are present, the immune response might sometimes respond negatively, making it difficult to use these fibers. Nanofibrous production is less dangerous than CNT production and poses less of a threat to the environment. It is fascinating that cerebral prostheses are created and produced using nanowires. In compared to nanostructured materials, alternative nano methods might not be capable of achieving the same functionalities (Modi et al., 2009; Naz et al., 2019).

### Polymeric nano micelles

Most of the most intriguing methodologies in nanotechnology are polymers microspheres. This arrangement is comprised a conceptual model with a buildings of polyethylene glycol in the core and a liposomes interior. The inclusion of hydrophobic toxic compounds is this program’s key benefit. The polymeric nanoparticles have sizes ranging between 10 and 100 nanometric scale (Castro et al., 2022; Romero-Ben et al., 2025).

### Polymeric nano liposomes

Lipoproteins called nano liposomes have a hydrophilic top and two hydrophobic cores. Their diameters range from 30 nm to a few meters. The phospholipid liposomes or the watery chambers of the liposomes can both hold a substantial quantity of medicines. Having an active component with changed interfaces can speed up bloodstream, minimize the likelihood that such lipid membranes will be eliminated by the hepatic, and diminish chemical peroxidation in serum. *In vitro* tests demonstrated their effectiveness for specific CNS dosage forms and demonstrated their extraordinary propensities for BBB drug translocation.

### Exosomes: New promising nanocarrier

A bulk of eukaryotic cells, including T and B lymphocytes, progenitor cells, and monocytes, produce macrovesicles, which are phospholipid relating to or characteristic extrinsic packets with micrometer sizes greater than 30–150 nm. Liposomes are exceptional and different from other solid lipid nanoparticles due to a number of unique characteristics. High cytocompatibility, nanoscale size, institutional and municipal cell communication abilities, minimal business consists, phenomenal capacity to overcome biological membranes, and significant capacity for stroma trying to target, entrap, and transport of a variety of subgroups of instability chemotherapeutic drugs, including fatty acids, hormone levels, peptides, and genetic mutations, have identified extracellular vesicles as viable chemical delivery vehicles for treating a variety of diseases, including cancer and myocardial infarction (Aryani and Denecke, 2016; Sarko and McKinney, 20172017; Niu et al., 2019; Zhang et al., 2019).

## Conclusion and future perspectives

CNS disorders are a big issue in today’s industrialized world. Nanotechnology has shown to be a cutting-edge and promising way for delivering drugs to the brain with pinpoint accuracy. However, in order to assess their dynamic behavior in biomedical science, we still need to learn more about their qualities and characteristics. For various CNS disorders that may be caused by multiple metabolic pathways, we now need a multimodal medicine. Nanodrugs could be the answer to this problem. Neurological issues can be linked to a variety of illnesses, including diabetes, trauma, and different psychotic conditions. As a result, nanomedicine requires the total eradication of these co-morbidity variables while limiting negative consequences. Apart from that, because genetic alterations in neuronal cells are difficult to achieve, nanotechnology-based drug delivery could be a viable treatment option for CNS illnesses. Although clinical trials using polymer-based gold nanoparticles and CNT nanomedicines are limited because to their strong physical and mechanical robustness, they may be helpful in transporting medications whose transit is still unclear. Despite the numerous benefits of nanoparticle-based therapy, there are still a number of difficulties to be resolved. There is no established method for evaluating the level of toxicity and targeted pharmaceutical release in the CNS at this time. As a result, current nanotechnology applications must be substantially developed in order to be safe and focused. Gold nanoparticle (US2011262546, US2011111040), lipid nanoparticle (WO2008024753, WO2008018932), chitosan nanoparticle (US2010260686), and SLN (US2011208161) are some of the nanomedicines that have registered for patents in complex CNS treatment in recent years. A growing population and a rise in brain illnesses necessitate the development of new promising remedies. Nanotechnology’s use in neuroscience will fill an unmet medical need and give sufferers hope. Nanomedicine of the next generation may be able to regulate prolonged and focused drug administration in a specific way. We still need to improve nanotechnological ways in pharmaceuticals for greater comprehension and improved living quality, rather than reducing side effects and increasing viability of nanodrugs. Nanomedicines’ potential use cannot be overstated, but their opportunity and risk formula also point to potentially dangerous adverse effects. Nanotechnology’s quick advancement in today’s research makes it hard to dismiss it merely on the basis of its disadvantages. Specific instructions must be followed in order to avoid the most harmful effects of nanotechnology. Nanotechnology-based drug delivery is also expected to revolutionize the timeframe for traditional drug distribution, with personalized pharmaceuticals significantly more efficient than the current standard. NPs are well-suited for the diagnosis and treatment of brain disorders due to their physical, chemical, and biological characteristics. The possibility of applying this technical technology to treat and diagnose CNS diseases has been promising. For improved medicine delivery to patients with CNS illnesses, several nanosolutions based on polymer-based techniques and nanoparticles are being investigated. To achieve development and hasten the treatment of illnesses affecting the CNS, experts in biomedical sciences (neuroscience, immunology, pharmacology, molecular imaging), materials science, biomaterials, and pharmaceuticals (polymers, nanomaterials, medicine, and genetics) are necessary. In both *in vitro* and *in vivo* models of malignant brain tumors, targeted delivery of chemotherapy drugs and antisense gene therapy has been made possible using nanocarriers, which has produced significant disease progression inhibition. Sadly, very few, if any, of this encouraging preclinical research have effectively been transferred to the clinic to affect patient care. Insufficient research on the toxicity of nanomaterials, their immunological compatibility, and the relative scarcity of *in vivo* trials are obstacles to this successful translation. In the future, nanomaterials might be utilized to replace a niche for transplanted stem cells, offering structural support as well as a sustained release of signaling molecules. In addition, the mechanical features of the nanomaterials as well as the incorporation of bioactive signals allow them to be functionalized to interact with stem cells. Collaboration across disciplinary boundaries will be essential to the effective application of nanotechnology in medicine. Although the use of nanotechnology alone is unlikely to be able to complete the challenging process of CNS repair, it does have a significant potential to influence clinical neuroscience treatment options. The most successful uses of nanoparticles in the treatment of CNS disorders have paired the effectiveness of nanoscale interventions with growth factors or cells that boost the overall effect of the treatment, emphasizing the significance of a combined approach to nanotherapeutics. Furthermore, improvements in our biology understanding of the mechanisms behind these illnesses can only increase the usefulness of nanotechnology applications in CNS disease. The aspect that has the potential to radically alter the practice of neurology is the interaction between new knowledge of the molecular causes of neurological illnesses and the multipurpose capabilities of nanotechnology. The severity of this condition and the current difficulties in AD neuropharmacology research are the main causes of the failure of CNS medication development. As a result, the future for their synergistic combination effect in CNS disease will be paved by the expansion in research of combinations of new nanocarriers and therapeutic targets. Although the development of nanomedicine with strong biodegradability and biocompatibility as a therapeutic agent for the treatment of CNS illnesses is a possibility, each NP has advantages and disadvantages of its own. As was previously mentioned, there are some negatives to nanomedicine that cannot be disregarded since they may buildup in the liver, kidney, and spleen and cause oxidative damage in the brain, which may be harmful to long-term health. In addition, there have been relatively few studies on the pharmacodynamics and pharmacokinetics of this nanomedicine, and their side effects continue to be a significant clinical concern.
