# Correction: Exploring the role of nanomedicines for the therapeutic approach of central nervous system dysfunction: at a glance

**DOI:** 10.3389/fcell.2026.1811452

**Published:** 2026-03-19

**Authors:** Md. Mominur Rhaman, Md. Rezaul Islam, Shopnil Akash, Mobasharah Mim, Md. Noor alam, Eugenie Nepovimova, Martin Valis, Kamil Kuca, Rohit Sharma

**Affiliations:** 1 Department of Pharmacy, Faculty of Allied Health Sciences, Daffodil International University, Dhaka, Bangladesh; 2 Department of Chemistry, Faculty of Science, University of Hradec Králové, Hradec Králové, Czechia; 3 Department of Neurology, Charles University in Prague, Faculty of Medicine in Hradec Králové and University Hospital, Hradec Králové, Czechia; 4 Andalusian Research Institute in Data Science and Computational Intelligence (DaSCI), University of Granada, Granada, Spain; 5 Department of Rasa Shastra and Bhaishajya Kalpana, Faculty of Ayurveda, Institute of Medical Sciences, Banaras Hindu University, Varanasi, India

**Keywords:** neurodegenerative diseases, blood-brain barrier, drug delivery, nanotechnology, nanomedicine and nanocarrier

The reference for “Vickers et al. (2017)” was erroneously written as “Vickers, N. J. J. C. b. (2017). Animal communication: When i’m calling you, will you answer too? Curr. Biol. 27, R713–R715. doi:10.1016/j.cub.2017.05.064”. It should be “Castro, K. C. D., Costa, J. M., and Campos, M. G. N. (2022). Drug-loaded polymeric nanoparticles: a review. *International Journal of Polymeric Materials and Polymeric Biomaterials*, *71*(1), 1-13, https://doi.org/10.1080/00914037.2020.1798436” and “Romero-Ben E, Goswami U, Soto-Cruz J, Mansoori-Kermani A, Mishra D, Martin-Saldaña S, Muñoz-Ugartemendia J, Sosnik A, Calderón M, Beloqui A, Larrañaga A. Polymer-based nanocarriers to transport therapeutic biomacromolecules across the blood-brain barrier. Acta Biomater. 2025 Apr; 196:17-49. doi: 10.1016/j.actbio.2025.02.065.”

The reference for “Fakruddin et al. (2012)” was erroneously written as “Fakruddin, M., Hossain, Z., and Afroz, H. J. J. o. n. (2012). Prospects and applications of nanobiotechnology: A medical perspective. J. Nanobiotechnology 10, 1–8. doi:10.1186/1477-3155-10-31”. It should be “Kumar, R., Aadil, K. R., Mondal, K., Mishra, Y. K., Oupicky, D., Ramakrishna, S., and Kaushik, A. (2022). Neurodegenerative disorders management: state-of-art and prospects of nano-biotechnology. *Critical reviews in biotechnology*, *42*(8), 1180-1212. https://doi.org/10.1080/07388551.2021.1993126.”

The reference for “Singh et al. (2021)” was erroneously written as “Singh, A. K., Singh, S. S., Rathore, A. S., Singh, S. P., Mishra, G., Awasthi, R., et al. (2021). Lipid-coated MCM-41 mesoporous silica nanoparticles loaded with berberine improved inhibition of acetylcholine esterase and amyloid formation. ACS Biomater. Sci. Eng. 7, 3737–3753. doi:10.1021/acsbiomaterials.1c00514”. It should be “Upadhyay, Ravi Kant. “Drug delivery systems, CNS protection, and the blood-brain barrier.” *BioMed research international* 2014, no. 1 (2014): 869269 https://doi.org/10.1155/2014/869269”.

The original article has been updated.

